# New Insights into Cooperative Binding of Homeodomain Transcription Factors PREP1 and PBX1 to DNA

**DOI:** 10.1038/srep40665

**Published:** 2017-01-17

**Authors:** Chiara Zucchelli, Elena Ferrari, Francesco Blasi, Giovanna Musco, Chiara Bruckmann

**Affiliations:** 1 Biomolecular NMR Unit, S. Raffaele Scientific Institute, via Olgettina 60, 20133, Milano, Italy; 2 IFOM (Foundation for Italian Cancer Research Institute of Molecular Oncology), via Adamello 16, 20139, Milan, Italy

## Abstract

PREP1 and PBX1 are homeodomain (HD) transcription factors that play crucial roles in embryonic development. Here, we present the first biophysical characterization of a PREP1 HD, and the NMR spectroscopic study of its DNA binding pocket. The data show that residues flanking the HD participate in DNA binding. The kinetic parameters for DNA binding of individual PREP1 and PBX1 HDs, and of their combination, show that isolated PREP1 and PBX1 HDs bind to DNA in a cooperative manner. A novel PREP1 motif, flanking the HD at the C-terminus, is required for cooperativity.

The 60-amino-acid-long homeodomain (HD) is one of the most important eukaryotic DNA-binding motifs, highly conserved in sequence, structure and mechanism of DNA binding^1^. The fold adopted by the HD has been studied in a number of high-resolution HD-DNA complex high-resolution structures solved by X-ray crystallography and NMR spectroscopy (for examples, see^2^,^3^,^4^,^5^). Despite the similarity in the structure of the DNA-binding motif, HD-containing proteins regulate many distinct biological processes. In particular, cell identities are controlled by the large family of HOX transcription factors^6^,^7^,^8^,^9^, while early embryonic development and tumorigenesis are controlled by the PBC and MEIS families^10^,^11^. PBX1 (PBC family), MEIS1 and PREP1 (MEIS family) belong to a class of HDs characterized by a three-amino-acid-loop-extension (TALE) between the first and second α-helix of the HD^12^. PREP1 (also known as PKNOX1) or MEIS1 form with PBX1, a stable DNA-independent heterodimer, through the PBC-A domain of PBX1 and the conserved N-terminal MEIS-A and MEIS-B domains of PREP1 or MEIS1^13^,^14^. PBX1:PREP1 and PBX1:MEIS1 pairs are necessary for subcellular localization and PBX1 stability^15^,^16^,^17^, while the presence of both HDs is required for DNA binding, as the proteins on their own show limited affinity for DNA^18^,^19^. In contrast to that of PREP/MEIS:PBX, HOX:PBX complex formation is DNA-dependent. HOX and PBX1 bind DNA cooperatively and their dimerization requires a highly conserved hexapeptide motif located N-terminal to the HOX HD, which contacts a pocket created by the TALE motif of PBX^3^,^9^,^20^,^21^,^22^,^23^. However, due to DNA conformability, Extradenticle (EDX; the Drosophila homolog of PBX1) retains some cooperativity with Ultrabitorax (Ubx) even after the removal of the Hox hexapeptide^23^. Recent evidence show an additional hexapeptide-independent mode of PBX recruitment through an additional motif, UbdA, located immediately C-terminal to the HOX HD^24^,^25^.

As shown in many crystal and NMR structures^3^,^21^,^26^, HD transcription factors contact DNA recognition sites through residues N_51_ and R_55_ in the third α-helix of the HD. N_51_ mediates the bidentate hydrogen bond with an adenine base, while R_55_ forms two hydrogen bonds with a guanine and both contact DNA in the major groove^3^. In addition to these hydrogen bonds, van der Waals interactions and water-mediated hydrogen bonds with the DNA sugar–phosphate backbone occur. Common ways to increase a transcription factor specificity for DNA involve interactions with the minor groove of DNA and/or cooperative partnerships with other transcription factors. For example, the binding of HD protein MATa1 with MATα2 depends on the 21-residues C-terminal tail of MATα2, located immediately after the HD^2^,^27^. The C-terminal tail of MATα2 undergoes a conformational change upon binding DNA in the presence of MATa1, thus becoming ordered, allowing contact to the MATa1 HD at a surface that does not participate in DNA binding^27^. It was also observed for HOX proteins that even non-specific interactions with DNA can increase DNA-binding affinity^21^.

PREP1 does not have a hexapeptide-like motif and strongly associates with PBX1 in the absence of DNA; therefore, the DNA-binding mechanisms of PREP1-PBX1 and PBX-HOX must be different. We have recently reported that DNA binding induces a conformational change in the full-length PBX1:PREP1 heterodimer^19^. Moreover, PBX1 and PREP1 can bind DNA independently when uncoupled, but require a specific functional interaction at the HD level for high affinity, thereby being sensitive to the DNA-binding state of the partner^19^. However, no further information is available on the dimerization and DNA binding activity of the PBX1-PREP1 combination at the HD level.

In this study, we have explored the functional and physical-chemical properties of isolated HDs and compared the affinity to DNA of different PREP1 HD constructs. We have quantified the ability of each of these HDs to bind DNA on their own and in combination. PREP1 HD, while specifically discriminating between different DNA motifs, binds DNA with low affinity. The presence of a preformed PBX1 HD:DNA complex significantly increases the DNA affinity of PREP1. Moreover, the binding properties of the PREP1 HD are different depending on the presence of C- and N-terminal flanking regions. The DNA interactions of two PREP1 HD constructs that differ in the amino- and carboxy-terminal extensions were studied by NMR spectroscopy. Indeed, the data show that the PREP1 HD carries important determinants for specificity and its flanking sequences are essential for formation of a high affinity complex with DNA. Most importantly, our data demonstrate that also PBX1:PREP1 HDs cooperate on DNA binding.

## Results

### Recombinant HDs

**Figure 1:**
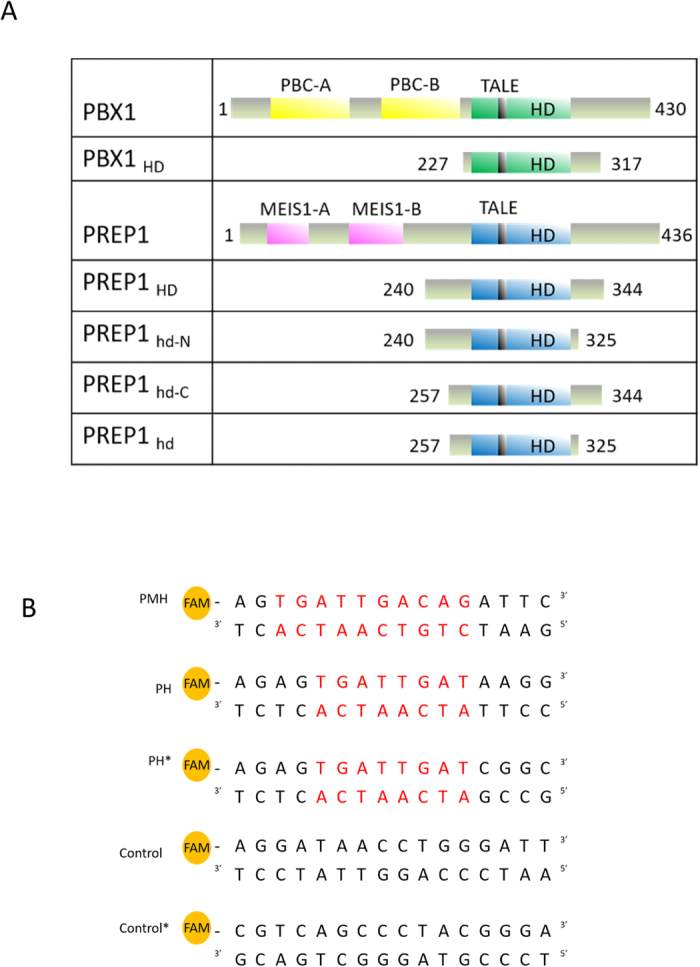
Panel A: Homeodomain (HD) protein constructs used for characterizations described in this study, in comparison with full-length proteins. PBX1_HD_ and HOXB1_HD_ constructs are the same used in the X-ray crystallographic study^3^. PREP1 HD is present in four lengths, PREP1_257-325_ (PREP1_hd_), PREP1_240-344_ (PREP1_HD_) and two intermediate lengths (PREP1_257-344_ - PREP1_hd-C_ - and PREP1_240-325_ -PREP1_hd-N_-), where respectively the N- or C-terminal extensions of the HD are omitted. Production of the proteins is described in the Materials and Methods section. PBC-A and PBC-B domains of PBX1 are those required for its dimerization with PREP1. TALE lies between helix 1 and helix 2 of the HD. MEIS-A and MEIS-B domains are PREP1 motifs required for heterodomerization with PBX1. Panel B: Consensus sequences for DNA binding by PREP1-PBX1: the very frequent decameric (PMH) and the less frequent octameric (PH) oligonucleotides^30^,^31^,^32^, are both highlighted in red in the sequence. Control probes correspond to two sequences that do not contain any PBX1 orPREP1 binding site. Oligonucleotides used for FP were 5′ labelled with 6-carboxyfluorescein (6-FAM) (see the Materials and Methods).

Purification of high quality individual PBX1 or PREP1 C-terminally truncated or full-length proteins was unsuccessful, as the single proteins, if not co-expressed, aggregate during the first purification step^28^. Isolated HDs of PREP1 and PBX1 were used and their interactions with DNA analyzed. Four different PREP1 HD constructs were cloned (Fig. 1A), the shortest (PREP1_hd_) consisted almost only of the HD (68 amino acids, from G_257_ to S_325_), and the longest (PREP1_HD_, from Q_240_ to F_344_) included residues from both N- and C-terminal sides. In addition, also two intermediate lengths, PREP1_257–344_ (PREP1_hd-C_) and PREP1_240–325_ (PREP1_hd-N_), were expressed. The _240_-QLQLQL-_245_ stretch in the N-terminal extension is computationally predicted by I-Tasser^29^ to adopt α-helical conformation; the C-terminal extends to residue 344, which corresponds to the break point of protein stability, as previously determined^28^. The PBX1_HD_ (residues 227–317) construct was identical to that previously used for X-ray crystallography studies^3^.

The recombinant proteins were purified by glutathione S-transferase (GST) affinity chromatography as previously described^28^, and the proteins used for electrophoretic mobility shift assay (EMSA), circular dichroism (CD), and NMR spectroscopy studies were further purified as described (see the Materials and Methods section). An SDS PAGE (polyacrylamide gel electrophoresis) of the four PREP1 HDs, after the first chromatographic step (GST affinity), is shown in Supplementary Figure S1A, while gel-filtration profiles and SDS PAGEs of purified PREP1_HD_ and PREP1_hd_ are shown in Figure S1B and C and show the degree of purity of the preparations.

### PREP1 HD C-terminal extension contains important determinant for DNA binding

The DNA sequence that was employed to measure binding to the HD, was based on ChIP-seq data on whole embryo trunk and several murine and human cell lines^30^,^31^,^32^,^33^. These studies have provided consensus sequences for DNA binding by PREP1-PBX1: the very frequent decameric (TGATTGACAG) and the less frequent octameric (TGATTGAT) oligonucleotides conform to the above consensus sequences. 5′-fluorescently labeled oligonucleotides (PMH, and PH) were used to measure the affinity of the individual HDs to DNA by fluorescence polarization (FP) and EMSA. A second form of the latter (PH*) corresponds to the sequence used to determine the X-ray structure of PBX1-HOXB1 HDs^3^,^30^, and aspecific sequences were used as control probes (Fig. 1B).

We first compared the affinities of the four PREP1-isolated HD constructs for the PREP1-PBX1 specific PMH, as determined by FP (see below). PREP1_HD_ has a binding affinity (*K*_D_) (15.4 μM) two-fold lower than that of PREP1_hd_ (31.8 μM) (Table 1). The C-terminal tail seems to contribute more significantly than the N-terminal tail, as the *K*_D_ of PREP1_hd-C_ (15.5 μM) is the same as that of PREP1_HD_ (Table 1), whereas the *K*_D_ of PREP1_hd-N_ (23.7 μM) is reduced. These results suggest that the presence of PREP1 HD C-terminal extension residues is important for high affinity DNA binding. Through FP, the PREP1_HD_ mutant was tested, in which K_331_, K_333_, K_334_, and K_335_, located in the C-terminal extension, were all mutated to alanine (PREP1_HD_^KKKK→AAAA^); lysine residues are positively charged and might be responsible for the higher affinity of PREP1_HD_ and PREP1_hd-C_ to the negatively charged DNA. Indeed, mutation of the four lysine residues increased the *K*_D_ to 21 μM, thereby suggesting that the contribution of the C-terminal tail is based in part on an electrostatic effect (Table 1).

### How isolated PREP1 and PBX1 HDs bind to different sequences

A comparison of the affinities reveals that PREP1_HD_ discriminates between binding sites (Table 1), exhibiting a preference for PMH (*K*_D_ 15.4 μM) with respect to PH (*K*_D_ 23.5 μM), and control probe (*K*_D_ 158.7 μM). PREP1_hd_ consistently shows a higher *K*_D_ with any of the DNA motifs compared to PREP1_HD_; this indicates a generally lower affinity for DNA.

PBX1_HD_ has a *K*_D_ for PMH (0.36 μM) 6-fold lower than for PH (2.1 μM) and the control oligo (2.5 μM), indicating that PBX1_HD_ has higher affinity but lower specificity for DNA. The same affinity (*K*_D_ 3.3 μM) is obtained with PH* the sequence of which is absolutely identical to that used in the crystal structure study^3^. The high affinity of PBX1_HD_ for the control oligo was confirmed also for a second control (control*, *K*_D_ 2.1 μM). The data are summarized in Table 1.

In EMSA, DNA probes (4 μM) were incubated with increasing amounts of HD, using protein:DNA ratios of 0.5 (protein at 2 μM), 1 (protein at 4 μM), and 2 (protein at 8 μM). To visualize DNA and proteins, the electrophoresis gels were stained with ethidium bromide and subsequently with coomassie blue.

**Figure 2:**
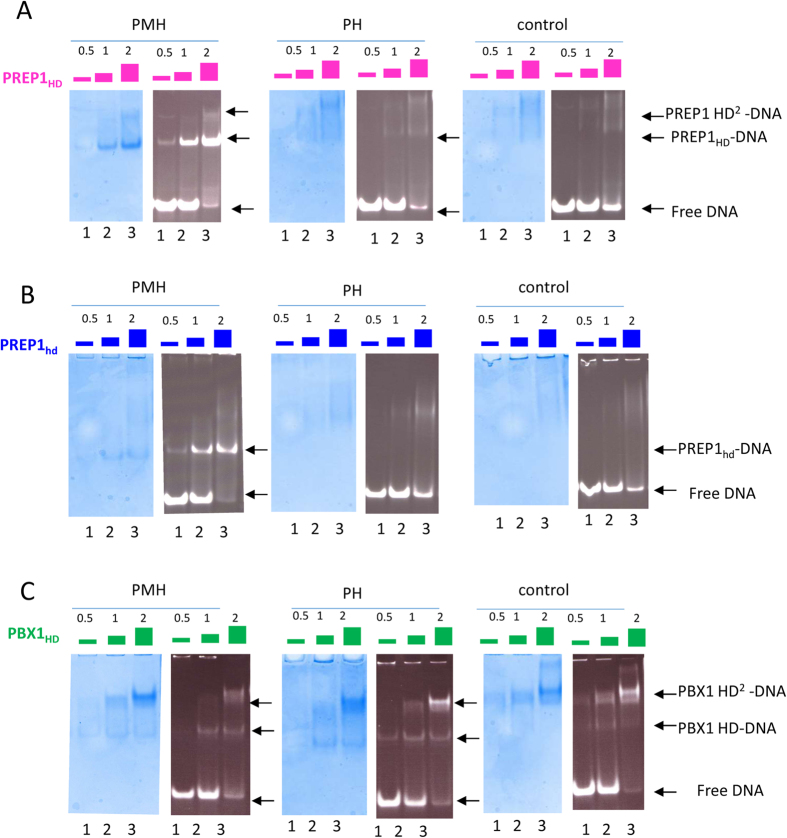
EMSA with single HDs. DNA probes were incubated with different amounts of HD. Protein:DNA ratios were 0.5 (DNA excess), 1 (equimolar ratio), or 2 (protein excess). Gels were stained both with ethidium bromide and coomassie blue to visualize DNA or proteins, respectively. Panel A: PREP1_HD_ forms two different complexes with PMH: the first is visible, at protein:DNA ratio of 0.5 (lanes 1 and 2, left panel), and the second as a smeared band, at protein:DNA ratio of 2 (lane 3, left panel). This slower migrating band might represent binding of two PREP1_HD_ proteins to DNA. In the case of PH (central panel) and control probes (right panel), a specific DNA complex is not formed. Panel B: PREP1_hd_ forms a single complex with PMH; whereas binding to control and PH oligos is weak and no specific bands are formed. Panel C: PBX1_HD_ forms a single complex with PMH and PH oligos at protein:DNA ratio of 0.5 (lane 1); by increasing the protein concentration, a slower migrating band becomes visible, possibly due to binding of a second monomer to DNA. PBX1 binds also the control oligo.

As shown in Fig. 2A, PREP1_HD_ forms two different complexes with PMH, the first is visible with all protein:DNA ratios (lanes 1–3 left panel of Fig. 2A), and the second, slower migrating diffused band, at a protein:DNA ratio of 2 (lanes 2, 3, left panel of Fig. 2A). The latter band likely represents binding of two PREP1_HD_ to DNA. In the cases of PH (central panel) and control probes (right panel), the bands tend to be diffused as if the complexes were less stable, dissociating while the gel is running. Nevertheless, the migration of the protein (coomassie staining) perfectly correlates with that of DNA (ethidium bromide staining).

PREP1_hd_ shows (Fig. 2B) the same behavior as PREP1_HD_ for PMH, but binding to the control oligo is absent, as no bands are evident.

In the case of PBX1_HD_ (Fig. 2C), a single complex with both PMH and PH is seen only at a protein:DNA ratio of 0.5 (lane 1), but at higher ratios, a slower migrating band becomes visible. As for PREP1, this extra band probably corresponds to binding of a second monomer to DNA. Such behavior in EMSA has been previously reported also for other DNA-binding proteins^34^. While PREP1_HD_ and PREP1_hd_ do not bind to the control probe, in the case of the PBX1_HD_ clear bands are formed, indicating little discrimination of PBX1_HD_ between sequences. This agrees with the results of the ChIP-seq analysis^30^,^31^, which failed to identify a real consensus motif for DNA sites bound uniquely by PBX1. In the literature examples of PBX1 consensus sequences are reported^35^,^36^. However, in those studies it has not been investigated whether PBX1 was binding in heterodimeric form.

Also PREP1_hd-C_ and PREP1_hd-N_ form a single complex with PMH oligo, as shown in the Supplementary Figures 2A and B.

### PREP1 and PBX1 HDs synergize on binding DNA

We determined by FP if PREP1_HD_ and PREP1_hd_ bind to a preformed PBX1_HD_:DNA complex. PBX1_HD_ (at the concentration of its *K*_D_ for PMH, 0.3 μM) and PMH (at 9 nM) were pre-mixed and then titrated with increasing concentrations of PREP1_HD_ or PREP1_hd_. In this case, the *K*_D_ of PREP1_HD_ and PREP1_hd_ were 1.9 μM and 5.1 μM, respectively (Table 2), both significantly lower than for the single titrations of PREP1_HD_ and PREP1_hd_ with PMH. As a negative control, we titrated PREP1_HD_ with the preformed HOXB1_HD_:PMH complex; in this case the *K*_D_ value remained within the range of affinity of PREP1_HD_ alone (Supplementary Figure S3C), in agreement with literature data showing that PREP1 does not bind HOXB1^37^. Thus, the combination with PBX1_HD_ specifically increases the DNA affinity for PMH of both PREP1_HD_ and PREP_hd_.

Through EMSA, the interactions of HD heterodimers with DNA were also investigated. The experiments were performed using a preformed HD:PMH complex (fixed molar ratio, ensuring that half of the DNA was free and half bound to the HD), titrated with increasing concentration of the second HD.

**Figure 3:**
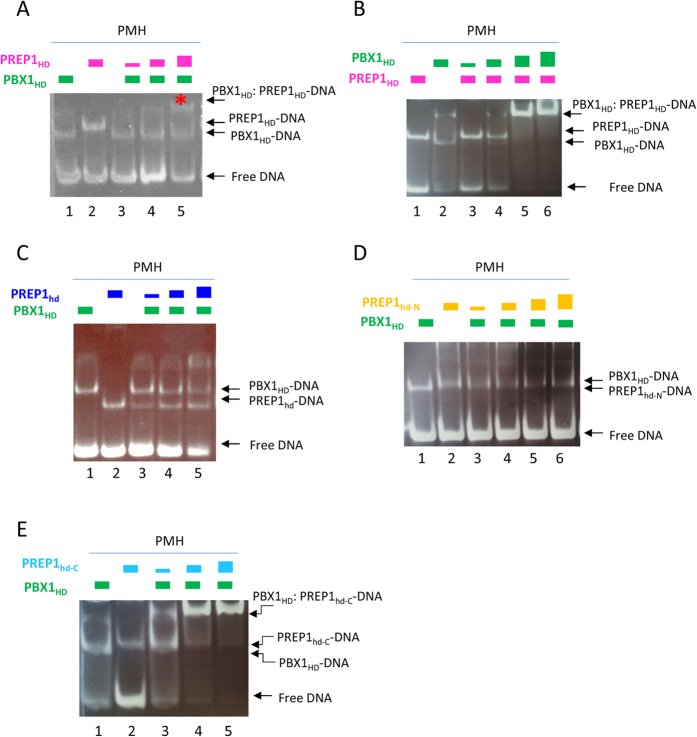
Evaluation of the cooperative binding between pairs of HDs. Panel A: A sample containing a preformed PBX1_HD_:PMH complex (at 4 and 8 μM, respectively) was titrated with increasing amounts of PREP1_HD_ (2, 4 and 8 μM). PREP1_HD_ binds preferentially to the PBX1_HD_:PMH complex rather than to the free DNA; the DNA pool decrease appreciably upon an increase in PREP1_HD_ concentration. At the lower PREP1_HD_ concentrations (lane 3) a slower migrating band is observed above the monomeric PREP1_HD_:DNA and PBX1_HD_:DNA complexes (identified in lanes 1 and 2, respectively). The band marked with a star in lane 5 was analyzed by mass spectrometry. Panel B: Titration of PREP1_HD_: PMH complex with PBX1_HD_. PREP1_HD_ (4 μM) and PMH (4 μM) were titrated with increasing concentrations of PBX1_hHD_ (2, 4 and 8 μM). Upon increase of PBX1_HD_ a slower migrating band appears, corresponding to PREP1_HD_:DNA:PBX1_HD_ complex. Panel C: Titration of PBX1_HD_: PMH complex with PREP1_hd_. PBX1_HD_ (4 μM) and PMH (4 μM) were titrated with increasing concentrations of PREP1_hd_ (2, 4 and 8 μM). Upon increase of PREP1_hd_ only a band corresponding to PREP1_hd_:DNA is visible, therefore, PREP1_hd_ in EMSA does not show clear binding to the preformed PBX1_HD_:DNA complex. Panel D: Titration of PMH:PBX1_HD_ with PREP1_hd-N_. PMH and PBX1_HD_ at a fixed concentration (8 μM and 4 μM, respectively) were titrated with increasing amounts of PREP1_hd-N_ (2, 4, 8, and 16 μM). Upon an increase in PREP1_hd-N_ no retarded band above the individual PMH:PREP1_hd-N_ and PMH:PBX11_HD_ complexes (lanes 3-5) are observed Panel E: Titration of PBX1_HD_:PMH with PREP1_hd-C_. PMH oligo and PBX1_HD_ at a fixed concentration (8 μM and 4 μM, respectively) were titrated with increasing concentrations of PREP1_hd-C_ (2, 4 and 8 μM). Upon an increase in PREP1_hd_-_C_, a slower migrating band corresponding to PREP1_hd-C_ :DNA: PBX1 _HD_ complex is visible.

The PBX1_HD_:PMH complex (protein at 2 μM, DNA at 4 μM) was titrated with PREP1_HD_ (2, 4, and 8 μM). Binding of PREP1_HD_ to the preformed PBX1_HD_:DNA complex is cooperative, as PREP1_HD_ preferentially binds the PBX1_HD_:DNA complex rather than free DNA; however, no PREP_HD_:DNA complex is visible. Already at low PREP1_HD_ concentrations (Fig. 3A, lanes 3 and 4) we observe a slower migrating band that runs above monomeric PREP1_HD_:DNA and PBX1_HD_:DNA complexes (lanes 1 and 2, respectively). Mass spectrometry analysis of this band (lane 5, indicated with an asterisk) confirmed the presence of both PREP1_HD_ and PBX1_HD_ (see Supplementary Figure S2C for the sequence coverage, while protein correspondence in National Center for Biotechnology Information (NCBI) database are shown in Table 3).

Likewise, in the converse experiment a PREP1_HD_:PBX1_HD_:DNA complex appears upon titration of the PREP1_HD_-DNA complex with PBX1_HD_ (Fig. 3B).

As a control, we titrated the HOXB1_HD_-PMH preformed complex with increasing amounts of PREP1_HD._ Again, a PREP1_HD_:HOXB1_HD_:DNA complex was not observed, as we could not visualize any retarded band (Supplementary Figure S3D). In conclusion, EMSA experiments, in agreement with FP, confirm that binding of PREP1_HD_ to the DNA site is relatively weak; it is clearly cooperative with PBX1 but not with HOXB1.

PREP1_hd_ does not show a clear cooperative behavior with the PBX1_HD_:PMH preformed complex in EMSA (Fig. 3C). Upon increase of PREP1_hd_, we observe only bands that correspond to PREP1_hd_:DNA or to PBX1 _HD_:DNA complexes. A weak slow-migrating band, more intense than the control no-PREP1_hd_ (lane 1), may be visible only at high concentration of PREP1_hd_ (lane 5). This result agrees with the FP data, where we observed a weak cooperative effect.

However, a clear cooperative effect is observed with PREP1_hd-C_ and not with PREP1_hd-N_, indicating that the sequences responsible for cooperative binding are located in the C-terminal extension (Fig. 3D,E).

### PREP_HD_ N-terminal and C-terminal extensions are mainly unstructured

**Figure 4:**
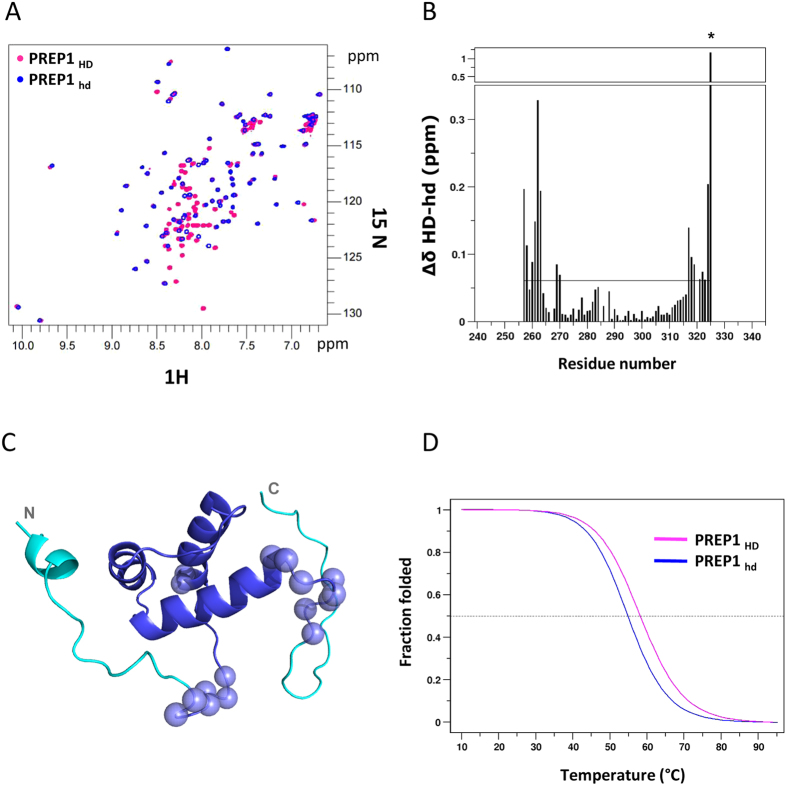
Panel A: Superposition of ^1^H-^15^N HSQC spectra of PREP1_HD_ (magenta) and PREP1_hd_ (blue). Panel B: Combined amide chemical shift difference (Δ*δ*) between PREP1_HD_ and PREP1_hd_ corresponding residues. Panel C: Cartoon representation of the PREP1_HD_ model: the residues (in blue) shared with PREP1_hd_, the N- and C- terminal extensions (in cyan) of PREP1_HD_. Spheres indicate PREP1_HD_ residues showing significant (>average) amide Δ*δ* with respect to PREP1_hd_. Panel D: Normalized CD melting curves for PREP1_HD_ (magenta) and PREP1_hd_ (blue) at 222 nm.

Structurally, PBX1 HD is well characterized, both in the presence and in the absence of DNA, and in complex or not with Hox proteins^3^,^38^,^39^,^40^. In order to have a structural rationale for the increased DNA binding affinity of PREP1_HD_, we investigated whether the N- and the C- terminal extensions of PREP1_HD_ were structured and if their presence influences/alters the HD structural core. Circular dichroism (CD) spectra (see Supplementary Figure S4) indicate that PREP1_HD_ does not have a higher content of α-helix with respect to PREP1_hd_, thereby suggesting that the N- and C- terminal extensions of PREP1_HD_ are unstructured. Moreover, superposition of the two-dimensional (2D) ^1^H-^15^N HSQC (heteronuclear single quantum correlation) NMR spectra of ^15^N-labeled PREP1_hd_ and PREP1_HD_ (Fig. 4A) reveals a high similarity between the two constructs. The majority of PREP1_HD_ resonances (magenta) almost coincide with those of PREP1_hd_ (blue), whereas amide resonances belonging to the N- and C-terminal extensions of PREP1_HD_ all cluster in the random coil region between 8.0 and 8.5 ppm on the ^1^H NMR axis. Residues located at the N-terminus (residues _242_-QLQL-_245_) have backbone chemical shift values suggestive of α-helical conformation (CSI = 1) (see Supplementary Figure S5). Accordingly, a chemical shift (^13^Cα, ^13^CO, ^13^Cβ, ^15^N, ^1^Hα, ^1^H_N_)-based three-dimensional (3D) model of PREP1_HD_ generated by CS23D2.0^41^ reveals that the N- and C-terminal extensions of PREP1_HD_ are unstructured, with the exception of a small helical turn involving residues _242_-QLQL-_245_ (see Supplementary Figure S5), in agreement with I-Tasser predictions. The systematic comparison of amide chemical shifts between PREP1_HD_ and PREP1_hd_ shows that the major differences are located not only around residues G_256_-S_257_ and S_324_-S_325_ (that correspond to N- and C-terminal residues of PREP1_hd_, but are internal in PREP1_HD_), but also in more internal regions (K_260_-R_263_, H_269_ and A_270_, I_317_-D_323_). This suggests that the presence of weak intramolecular interactions between the N- and C-terminal extensions and the HD core (Fig. 4B,C). In agreement with this hypothesis, we observe by CD an approximate 4 °C increase in the melting temperature (*T*_m_ = 58.24 °C) of PREP1_HD_ compared to PREP1_hd_ (*T*_m_ = 54.86 °C), as shown in Fig. 4D. In conclusion, a comparison of the CD and ^1^H-^15^N HSQC spectra of both constructs indicates on the one hand that the N- and C-terminal extensions of PREP1_HD_ are mainly unstructured, but on the other hand suggests that the N- and C- terminal extensions weakly interact with the HD structural core. This was assessed by the increased PREP1_HD_ *T*_m_ and by the chemical shift differences observed within the HD structured region.

### NMR titration of ^15^N PREP1_HD_ and ^15^N PREP1_hd_ with DNA

**Figure 5:**
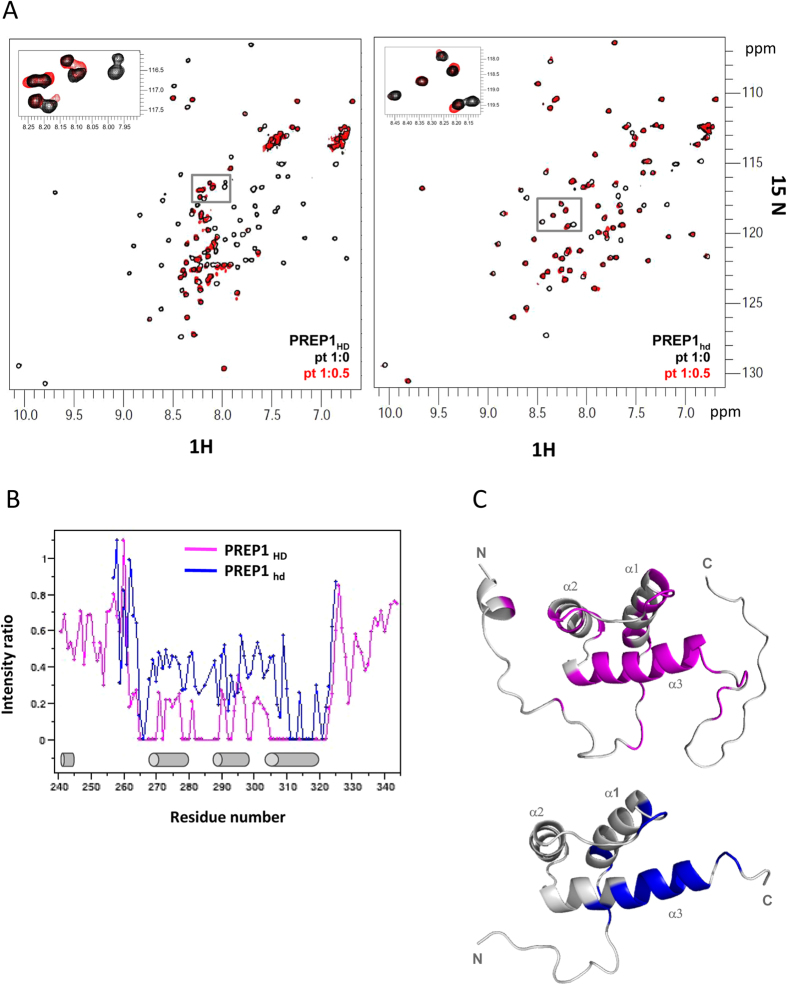
NMR titration of ^15^N-labeled PREP1_hd_ and PREP1_HD_ with PMH DNA oligo. Data reported correspond to a protein:PMH molar ratio of 1:0.5. Panel A: Superposition of ^1^H-^15^N HSQC spectra of free (black) and PMH-bound (red) PREP1_HD_. (left) and PREP1_hd_ (right) Panel B: Plot showing the peaks intensity reduction observed upon PMH binding to PREP1_hd_ and PREP1_HD_. Panel C: Residues disappearing or showing significant amide chemical shift displacement (as reported in the CSD plot in Supplementary Figure S6) are highlighted on PREP1_HD_ structural model (top, generated using CS23D2.0 web server) and PREP1_hd_ NMR structure (bottom, 1 × 2 N.pdb,).

We next investigated the interaction of DNA oligo PMH with ^15^N-labeled PREP1_HD_ and PREP1_hd_ using chemical shift perturbation, a very sensitive tool to detect residues that directly interact with a ligand or are indirectly affected by binding. Upon addition of substoichiometric quantities of unlabelled PMH to both ^15^N-labeled PREP1_HD_ and PREP1_hd_ we observe small chemical shift displacements (CSD) and significant disappearance and/or intensity reduction of several amide peaks in the ^1^H-^15^N HSQC spectra, mainly affecting the three helices of the HD (Fig. 5B and Figures S5, S6, and S7). This behavior is indicative of binding in the intermediate exchange regime on the NMR chemical shift time scale, compatible with dissociation constants in the low micromolar range, as measured by FP (Table 1).

Already at substoichiometric ratio, in both constructs peak disappearance is mostly evident for the third α-helix of the HD, involving residues _312_-NARRRIL-_318_ that are fundamental for DNA interaction^19^,^21^,^42^. Comparison of the peak intensity reduction and/or disappearance occurring in PREP1_HD_ and PREP1_hd_, clearly shows that the broadening effect is much more pronounced in PREP1_HD_ than in PREP1_hd_ (Fig. 5B), in agreement with its higher affinity for DNA. The N- and C-terminal extensions of PREP1_HD_ (i.e., residues preceding G_257_ and following S_325_, respectively) also experience a clear intensity reduction, thereby suggesting their involvement in binding. As a matter of fact, significant amide CSD were also observed for residues located on the N- and C-terminal tails of PREP1_HD_ before the first α-helix (L_245_, Q_254_, K_262_, R_263_) and just after the third α-helix (S_324_, S_325_) (see Supplementary Figure S8).

In order to have a 3D visualization of the DNA binding pocket, we have highlighted on the NMR 3D models of PREP1_hd_ and PREP1_HD_, those residues experiencing peak disappearance or significant CSD upon DNA interaction (CSD > average + standard deviation) (Fig. 5C).

Collectively these data confirm that the third α-helix is important for DNA binding and suggest that the N- and C-terminal extensions of PREP_HD_ also contribute to the interaction, possibly increasing the affinity.

## Discussion

Transcription factor activity depends on specific interactions between amino acids and critical DNA bases, as well as on interactions with other protein partners. PREP1 binds DNA through its highly-conserved HD, well separated from the PBX1-dimerization domain. Until now, the high-affinity binding to DNA has been solely attributed to the dimerization of PBX1 and PREP1 at the N-terminus. Here, we have shown that the isolated PREP1 HD binding to DNA involves also residues outside of the HD core. Although the measured affinities do not apply *in vivo*, where the full-length proteins and not just the HDs are present, this work represents the first biochemical and biophysical characterization of the isolated PREP1 HD.

1. As already demonstrated by several high-resolution structures^3^,^21^,^26^. mutational analysis^19^ and on the basis of studies of other HDs^43^, the positively charged N_312_-L_318_ stretch in the third helix of the HD contacts directly DNA. In agreement with these findings, the backbone amide peaks that correspond to these residues undergo substantial peak intensity reduction upon addition of DNA at substoichiometric concentrations in 2D ^1^H^15^N HSQC experiments.

2. Both the N- and C-terminal regions adjacent to the PREP1 _HD_ core are unstructured (except for a small helical turn involving residues _242_-QLQL-_245_). However, they stabilize the core HD structure increasing the *T*_m_ by 4 °C and promote PREP1 HD binding to DNA increasing affinity.

3. Indeed, the 2D ^1^H^15^N HSQC peak intensity reduction upon DNA binding is more pronounced in PREP1_HD_ compared to that of PREP1_hd_, and this suggests that the N- and C-terminal extensions increase DNA affinity, in agreement with EMSA (Fig. 3) and FP (Table 1) experiments. The NMR peaks corresponding to residues located in the N- and C- terminal extensions (such as Q_254_, K_262_, R_263_ before the first α-helix, and S_324_, S_325_ after the third α-helix) decrease their intensity in the presence of DNA, supporting this interpretation. Furthermore, mutation of four lysine residues (K_331_, K_333_, K_334_, and K_335_) (Table 1) in the electropositive patch of the C-terminal extension decreases DNA affinity, suggesting that a charge component contribution to the binding. Conceivably, the residues flanking the PREP1 HD core may not be directly involved in DNA binding, but can stabilize the three DNA-bound α-helices. In this model, the regions flanking PREP1 HD may contribute to DNA recognition, restricting the possible conformations of the DNA-binding domain thus favoring a unique interaction with DNA.

Previous studies have reported that the affinity for DNA of the full-length complex is in the low-nanomolar range^19^. This suggests that the interaction between the N-terminal regions is responsible for the profound changes in the DNA-binding domain activity. In addition, conformational changes and flexibility of the PBX1:PREP1 complex favor the robustness of the protein-DNA interaction, as deletion of the poly-alanine linker of PBX1 decreases 150-fold the affinity of the whole complex^19^. Now we demonstrate that the presence of PBX1_HD_ enhances the ability of PREP1_HD_ to bind DNA, with an 8-fold decrease in the *K*_D_. The preference for a preformed PBX1_HD_:DNA complex over free DNA is well visible in EMSA assays, where already at very low molar ratios PREP1_HD_ binds to PBX1_HD_:DNA, rather than to free DNA, with no detectable intermediates. Mass spectrometry analysis of the slower migrating band (Fig. 3A) confirms the presence of both PBX1_HD_ and PREP1_HD_ with high sequence coverage. Cooperativity is specific for PBX1_HD_ as HOXB1_HD_ does not cooperate with PREP1 (data shown in Supplementary Information).

When PBX1 and PREP1 HD contact DNA, the binding state of one determines the DNA preference of the other, as already observed by mutational studies of key residues in the third α-helix of PBX1 and PREP1 HDs^19^.

Our fluorescence and EMSA binding data suggest that the most likely mechanism mediating cooperativity is the presence of an uncharacterized motif, namely, the hexapeptide in Hox, which is involved in the dimerization of the HDs. In this respect, a region flanking the PREP1 HD at the C terminus, although mainly unstructured, appears to be important to enhance the cooperativity.

However, in the absence of an X-ray crystal structure, we cannot exclude other mechanisms: for example, cooperativity may be mediated by a conformational change in the DNA or by overlapping contacts of PBX1 and PREP1 with DNA^23^,^34^,^44^,^45^,^46^.

PREP1 on its own binds DNA weakly, but its affinity for the specific site increases when combined with PBX1. This *in vitro* cooperativity of PREP1 and PBX1 might result in a precise organization of transcriptional regulation, in which PREP1 contributes to selectivity and PBX1 to affinity.

## Materials and Methods

### Cloning of recombinant protein**s**

HDs proteins, listed in Fig. 1A (PREP1, PBX1) were all subcloned in a pGEX-6P vector (GenBank: KM817768) using BamHI/XhoI cloning sites. Primers used for cloning are listed in Table S1. The DNA template of PREP1 HD in which the four residues K_231_, K_233_, K_234_, and K_235_ were mutated to alanine was purchased from Genscript Corporation (Piscataway, NJ) and showed the following sequence:

5′CAGCTTCAGTTACAGTTAAACCAAGATCTCAGCATCTTGCATCAAGATGATGGTTCATCTAAGAACAAGAGGGGCGTCCTGCCAAAGCATGCCACGAACGTGATGCGGTCCTGGCTCTTCCAGCACATCGGGCATCCCTACCCAACAGAGGATGAGAAAAAACAGATTGCTGCTCAGACAAATTTGACACTACTCCAAGTCAACAACTGGTTCATCAATGCCAGAAGACGAATTCTTCAGCCAATGTTGGATTCAAGTTGTTCAGAGACCCCCGCAACAGCGGCAGCAACTGCTCAGAACCGGCCAGTTCAGAGG 3′.

### Protein expression and purification

Recombinant proteins were expressed and purified as described in the literature^28^. Expression was performed in BL21(DE3)pLysS *E. coli* strain (Promega, Madison, WI). Uniformly ^15^N- and ^13^C-^15^N-labelled PREP1_HD_ and PREP1_hd_ were expressed by growing *E. coli* BL21(DE3)pLysS cells (Promega) in minimal bacterial medium containing ^15^NH_4_Cl, with or without ^13^C-D-glucose (both from CortecNet, Voisins-le-Bretonneux, France). Protein expression was induced with 0.1 mM isopropyl-β-D-thiogalactopyranoside (IPTG) for 16–20 h at 16 °C. Cells were harvested by centrifugation and resuspended in lysis buffer (20 mM Tris-HCl pH 7.4, 1 M NaCl, 10% glycerol, 0.5 mM EDTA (Ethylenediaminetetraacetic acid) and 1 mM DTT (Dithiothreitol)) supplemented with Protease Inhibitor Cocktail Set III Calbiochem (Billerica, MA). Sonication was done with a Bandelin Sonopuls (Berlin, Germany) sonicator for 3 × 45 seconds with 5 pulses at 40% of max power. After sonication, bacterial lysates were cleared by centrifugation at 40,000 × g for 1 hour. Proteins were purified using glutathione-sepharose 4B beads (GE Healthcare, Milano, Italy) according to manufacturer’s instructions. GST was cleaved off with 10 μg/ml of 3C- preScission protease (GE Healthcare) for 16 hours at 4 °C. GST-free proteins were diluted in buffer (20 mM Tris pH 7.4, 10% glycerol, 0,5 mM EDTA, 0,5 mM EGTA and 1 mM Dithiotheitol) to a final 0.1 M NaCl concentration and purified on a Resource S (GE Healthcare) cation exchange column using a 0.1–1.0 M NaCl gradient for elution. The recombinant proteins used for EMSA, CD, or NMR spectroscopic experiments (PBX1, PREP1_HD_ and PREP1_hd_ HDs) were further purified by size-exclusion chromatography on a Superose 6 10/300 column (GE Healthcare) equilibrated in 20 mM Na_2_HPO4/NaH_2_PO4 pH 7.2, 150 mM NaCl, 5% glycerol, and 1 mM DTT at a flow rate of 0.3 ml/min. Protein markers used for size-exclusion chromatography were the gel filtration standards from Bio-Rad (Hercules, CA). Protein concentrations were determined by the UV absorption at 280 nm. The extinction coefficients of the proteins are reported in Supplementary Table S2, and they were calculated using the online tool ProtParam^47^.

### DNA oligonucleotides

DNA oligonucleotides for EMSA, NMR and CD experiments were unlabeled, however, those used in FP were 5′-labeled with 6-FAM (6-Carboxyfluorescein) dye. They were purchased from Sigma and purified by HPLC by the manufacturer. 0.1–1 mM double-strand DNA oligonucleotides were prepared by annealing equimolar concentration of each strand in 10 mM Tris-HCl pH 8.0, 50 mM NaCl, 1 mM EDTA. This mixture was heated to 95 °C for 5–10 minutes and slowly cooled to room temperature. The sequences of the DNA motifs are shown in Fig. 1B; for NMR spectroscopic characterization we used the DNA oligo PMH.

### Fluorescence polarization

Serial dilutions of the protein were performed in 20 mM Na/K phosphate pH 7.2, 150 mM NaCl, 5% glycerol and 1 mM TCEP (Tris(2-carboxyethyl) phosphine). 20 μl of the protein solutions were transferred in a microplate and mixed with a fixed volume of 6-FAM-DNA (9 nM as final concentration). Binding reactions were incubated to reach steady state equilibrium at room temperature, in the dark, for 30 minutes. Fluorescence polarization assays were performed in a 20 μl final volume in flat bottom, black plates (Corning® Low Volume 384 Well Black Flat Bottom Polystyrene NBS™ Microplate). Saturation binding isotherms were generated at a fixed concentration of DNA with increasing concentrations of the proteins (from 0.1 to 60.0 μM) in 20 mM Na/K phosphate pH 7.2, 150 mM NaCl, 5% glycerol and 1 mM TCEP. *K*_D_ values were calculated using non-linear Michaelis*-*Menten fitting*s*. Readings were acquired on a Tecan Infinite F200 fluorimeter, with an excitation filter at 485 nm (20 nm bandwidth) and emission filter at 535 nm (25 nm bandwidth). Data were fitted with GraphPad Prism (http://www.graphpad.com/scientific-software/prism). One-site Michaelis-Menten binding model best fitted the experimental curves. All the experiments were performed in triplicate and the final *K*_D_ values reported in the tables correspond to the average of three independent experiments.

### EMSA

Non-denaturing gels were prepared in a final volume of 15 ml with 10% Acrylamide:bisacrylamide solution (37.5:1), 0.8% glycerol, 0.5 × TBE (Tris/Borate/EDTA, 45 mM Tris base, 45 mM boric acid, 1 mM EDTA pH 8.3), 0.1% APS (Ammonium Persulfate) and 6 μl TEMED (Tetramethylethylenediamine). Binding reactions were assembled at room temperature in a total volume of 15 μl (in 20 mM Na/K Phosphate pH 7.2, 150 mM NaCl, 5% glycerol, 1 mM TCEP) and incubated 10–15 minutes before loading on non-denaturing 10% acrylamide gel in running buffer 0.5 × TBE. The gel was pre-electrophoresed, at 4 °C, for 20 minutes at 90 V. Electrophoretic running continued for 50 minutes at 4 °C. The gel was stained for 10 minutes in ethidium bromide (diluted 1:10000) in 50 ml of 0.5 × TBE, for DNA detection. Then the gel was stained in coomassie staining for protein detection. Poly (dI/dC) were purchased from Roche Diagnostics S.p.A. (Monza, Italy).

### Mass spectrometry

The bands of interest were cut from gels and trypsinized as described^48^. Peptides were desalted as previously described^49^, dried in a Speed- Vac and resuspended in 7 μl of 0.1% formic acid reversed-phase capillary chromatography/electrospray ionization-mass spectrometry (LC-ESI-MS/MS) of 5 μL of each sample was performed on a Fourier transformed-LTQ mass spectrometer (FT-LTQ) (Thermo Electron, San Jose, CA). Peptide separation was achieved through a linear gradient from 100% solvent A (5% acetonitrile, 0.1% formic acid) to 20% solvent B (60% acetonitrile, 0.1% formic acid) over 20 min and from 20% to 80% solvent B in 5 min at a constant 0.3 μL/min flow rate on Agilent chromatographic separation system 1100 (Agilent Technologies, Waldbronn, Germany). The liquid chromatography system was connected to a 10.5 cm fused-silica emitter of 100 μm inner diameter (New Objective, Inc. Woburn, MA USA), packed in-house with ReproSil-Pur C18-AQ 3 μm beads (Dr. A. Maisch Gmbh, Ammerbuch, Germany) using a high-pressure bomb loader (Proxeon, Odense, Denmark).

Data acquisition mode was set to obtain one MS scan followed by five MS/MS scans of the five most intense ions in each MS scan. MS/MS spectra were limited to one scan per precursor ion followed by 1 min of exclusion. The mascot generic format (MGF) files were extracted using DATA SuperCharge (v.1.19, www.cebi.sdu.dk) while Database search was performed using Mascot Daemon v.2.3.2 set up with the following parameters: Database NCBInr, Taxonomy Homo Sapiens, enzyme Trypsin, Max missing cleavage 2, fixed modification carbamidomethyl (C), variable modification oxidation (M), peptide tolerance 10 ppm, MS/MS tolerance 0.5 Da, Instrument ESI-TRAP.

### Circular Dichroism spectroscopy

CD spectra were acquired on a Jasco J-815 CD spectrometer at 20 °C, from 200 to 260 nm. Typical protein concentrations were 10 μM in 20 mM Na_2_HPO_4_/NaH_2_PO_4_ pH 7.2 and 150 mM NaF. Spectra were averaged over 4 scans and corrected by subtracting the buffer spectrum and smoothed. The observed ellipticity *θ* (mdeg) was converted into molar residue ellipticity (MRE, deg cm^2^ dmol^−1^). The same samples were used for thermal denaturation experiments, performed recording the *θ* values at 222 nm, from 10 °C to 96 °C, with 1 °C intervals, 1 °C/min rate, average time 0.5 sec and bandwidth 2 nm. The *T*_m_ was calculated with non-linear curve fitting of the equation:


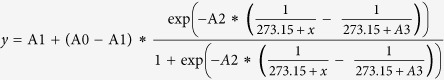


where A0 and A1 are *θ* at the unfolded and folded states, respectively, A2 is Δ*H*/*R* (set to 10^5^ as initial guess) and A3 is *T*_m_.

### NMR spectroscopy

NMR experiments were performed at 28 °C on a Bruker Avance 600 MHz spectrometer equipped with TCI cryoprobe and pulsed field gradients. Data were processed with TopSpin 3.2 (Bruker) and analyzed using CcpNmr Analysis 2.1.5^50^. All the samples were dissolved in the same NMR buffer (20 mM Na/K phosphate buffer pH 6.0, 150 mM NaCl, 1 mM DTT, 5% glycerol, 5% D_2_O, 0.3 mM 4,4-dimethyl-4-silapentane-1-sulfonic acid (DSS)). Backbone (^1^H_N_, ^15^N, ^13^Ca,^13^CO) and ^13^C side chain resonances of PREP1_HD_ recombinant protein were assigned through 2D and 3D ^1^H-^15^N HSQC, ^1^H-^13^C HSQC, HNCA, CBCA(CO)NH and CC(CO)NH NMR experiments, acquired at 28 °C on a 0.43 mM ^15^N-^13^C- labeled PREP1_HD_ sample. As the ^1^H-^15^N HSQC spectra of ^15^N-labeled PREP1_hd_ and PREP1_HD_ constructs are almost super-imposable, PREP1_hd_^1^H_N_ and ^15^N chemical shifts were assigned based on PREP1_HD_ assignment. NMR titrations were performed with stepwise additions of the DNA oligo PMH (8.1 mM) into ^15^N-labeled PREP1_HD_ or PREP1_hd_ (0.1 mM) up to a 1.5 molar excess. At each titration point, 1D ^1^H and 2D ^1^H-^15^N HSQC spectra were acquired. The weighted average of the ^1^H_N_ and ^15^N chemical shifts displacements (CSD) upon binding was calculated as CSD = [(Δ_HN_^2^+Δ_N_^2^/25)/2]^1/2^ 51. We considered residues showing CSD > average+*σ*_0_ as significantly affected upon DNA interaction. The *σ*_0_ value was calculated excluding any residue for which the CSD value was bigger than 3*σ*; recalculating *σ* and iterating these calculations until no further residues were excluded. This procedure has been used to avoid biasing the distribution by including the small number of residues with large CSDs^51^.

For each titration point and for each protein residue, we also calculated the decrease in peak intensity (*I*) as *I*/*I*_o_, where *I*_o_ is the intensity of the peak in the free protein.

### Secondary Structure and 3D Model of PREP1_HD_

The secondary structure elements were identified with CSI 3.0 server (http://csi3.wishartlab.com/cgi-bin/index.php)^52^ based on PREP1_HD_^13^Cα, ^13^CO, ^13^Cβ, ^15^N, ^1^Hα, ^1^H_N_ chemical shifts. Chemical shifts were also used as input to generate a 3D model of PREP1_HD_ using CS23D2.0 web server (http://www.cs23d.ca/index.php)^41^.

## Additional Information

**How to cite this article**: Zucchelli, C. *et al*. New Insights into Cooperative Binding of Homeodomain Transcription Factors PREP1 and PBX1 to DNA. *Sci. Rep.* **7**, 40665; doi: 10.1038/srep40665 (2017).

**Publisher's note:** Springer Nature remains neutral with regard to jurisdictional claims in published maps and institutional affiliations.

## Supplementary Material

Supplementary Information and Figures

## Figures and Tables

**Table 1:** *K*_D_ values for individual HDs with different DNA sequences, measured by FP.

	*K*_D_ (μM)		
	PMH	PH	Control
PREP1_HD_^KKKK→AAAA^	21.8 ± 0.8	—	—
PREP1_hd-N_	23.7 ± 2.6	—	—
PREP1_hd-C_	15.5 ± 0.6	—	—
PREP1_HD_	15.4 ± 1.0	23.5 ± 2.0	158.7 ± 5.2
PREP1_hd_	31.8 ± 1.5	51.4 ± 2.5	836.5 ± 51.8
PBX1_HD_	0.36 ± 0.1	PH: 2.1 ± 0.3PH*: 3.3 ± 0.5	Control: 2.5 ± 0.4Control*: 2.1 ± 0.1

The values are the average of three separate experiments (*n* = 3), each run in triplicate.

**Table 2:** FP *K*_D_ measurement of PREP1 HDs in the presence of a preformed PBX1_HD_: DNA complex.

Proteins	*K*_D_ (μM)
PREP1_HD_ + PBX1_HD_: PMH oligo	1.9 ± 0.5
PREP1_hd_ + PBX1_HD_: PMH oligo	5.1 ± 1.8

DNA sequence used: PMH (binding site of PBX1:PREP1).

The values are the average of three separate experiments (*n* = 3), each run in triplicate.

**Table 3:** Mass spectrometry analysis of EMSA titrations bands from Fig. 3.

Gi	Protein	Identified peptides	Mascot score
gi|107390	PBX1	4	316
gi|2052385	PREP1	4	172

The slow-migrating band indicated with red asterisk in Fig. 3A was cut and analyzed by mass spectrometry (see Materials and Methods). There, peptides belonging both to PREP1 and PBX1 HDs have been identified (Gi, sequence identification number); the number of unique identified peptides and their relative Mascot score are shown for each protein.

## References

[b1] Burglin T. R. & Affolter M. Homeodomain proteins: an update. Chromosoma 125, 497–521 (2016). 26464018 10.1007/s00412-015-0543-8 PMC4901127

[b2] Li T., Jin Y., Vershon A. K. & Wolberger C. Crystal structure of the MATa1/MATα2 homeodomain heterodimer in complex with DNA containing an A-tract. Nucleic Acids Res. 26, 5707–5718 (1998). 9838003 10.1093/nar/26.24.5707 PMC148023

[b3] Piper D. E., Batchelor A. H., Chang C. P., Cleary M. L. & Wolberger C. Structure of a HoxB1-Pbx1 heterodimer bound to DNA: role of the hexapeptide and a fourth homeodomain helix in complex formation. Cell 96, 587–597 (1999). 10052460 10.1016/s0092-8674(00)80662-5

[b4] Pradhan L. . Crystal Structure of the Human NKX2.5 Homeodomain in Complex with DNA Target. Biochemistry (Mosc.) 51, 6312–6319 (2012). 10.1021/bi300849c PMC3448007 22849347

[b5] Wilson D. S., Guenther B., Desplan C. & Kuriyan J. High resolution crystal structure of a paired (Pax) class cooperative homeodomain dimer on DNA. Cell 82, 709–719 (1995). 7671301 10.1016/0092-8674(95)90468-9

[b6] Catela C., Shin M. M., Lee D. H., Liu J.-P. & Dasen J. S. Hox Proteins Coordinate Motor Neuron Differentiation and Connectivity Programs through Ret/Gfralpha Genes. Cell Rep. doi: 10.1016/j.celrep.2016.01.067 (2016). PMC4775310 26904955

[b7] Chen J. Y. . Hoxb5 marks long-term haematopoietic stem cells and reveals a homogenous perivascular niche. Nature 530, 223–227 (2016). 26863982 10.1038/nature16943 PMC4854608

[b8] Merabet S. & Galliot B. The TALE face of Hox proteins in animal evolution. Front. Genet. 6, 267 (2015). 26347770 10.3389/fgene.2015.00267 PMC4539518

[b9] Moens C. B. & Selleri L. Hox cofactors in vertebrate development. Dev. Biol. 291 (2006). 10.1016/j.ydbio.2005.10.032 16515781

[b10] Dardaei L., Longobardi E. & Blasi F. Prep1 and Meis1 competition for Pbx1 binding regulates protein stability and tumorigenesis. Proc. Natl. Acad. Sci. USA 111 (2014). 10.1073/pnas.1321200111 PMC3956142 24578510

[b11] Longobardi E. . Prep1 (pKnox1)-deficiency leads to spontaneous tumor development in mice and accelerates EmuMyc lymphomagenesis: a tumor suppressor role for Prep1. Mol. Oncol. 4, 126–134 (2010). 20106730 10.1016/j.molonc.2010.01.001 PMC5527898

[b12] Mukherjee K. & Burglin T. R. Comprehensive analysis of animal TALE homeobox genes: new conserved motifs and cases of accelerated evolution. J. Mol. Evol. 65, 137–153 (2007). 17665086 10.1007/s00239-006-0023-0

[b13] Berthelsen J., Zappavigna V., Mavilio F. & Blasi F. Prep1, a novel functional partner of Pbx proteins. EMBO J. 17, 1423–1433 (1998). 9482739 10.1093/emboj/17.5.1423 PMC1170490

[b14] Chang C. P. . Meis proteins are major *in vivo* DNA binding partners for wild-type but not chimeric Pbx proteins. Mol. Cell. Biol. 17, 5679–5687 (1997). 9315626 10.1128/mcb.17.10.5679 PMC232416

[b15] Berthelsen J., Zappavigna V., Ferretti E., Mavilio F. & Blasi F. The novel homeoprotein Prep1 modulates Pbx-Hox protein cooperativity. EMBO J. 17, 1434–1445 (1998). 9482740 10.1093/emboj/17.5.1434 PMC1170491

[b16] Berthelsen J., Kilstrup-Nielsen C., Blasi F., Mavilio F. & Zappavigna V. The subcellular localization of PBX1 and EXD proteins depends on nuclear import and export signals and is modulated by association with PREP1 and HTH. Genes Dev. 13, 946–953 (1999). 10215622 10.1101/gad.13.8.946 PMC316640

[b17] Ryoo H. D., Marty T., Casares F., Affolter M. & Mann R. S. Regulation of Hox target genes by a DNA bound homothorax/Hox/extradenticle complex. Development 126, 5137–5148 (1999). 10529430 10.1242/dev.126.22.5137

[b18] Knoepfler P. S., Calvo K. R., Chen H., Antonarakis S. E. & Kamps M. P. Meis1 and pKnox1 bind DNA cooperatively with Pbx1 utilizing an interaction surface disrupted in oncoprotein E2a-Pbx1. Proc. Natl. Acad. Sci. USA 94, 14553–14558 (1997). 9405651 10.1073/pnas.94.26.14553 PMC25052

[b19] Mathiasen L. . The flexibility of a homeodomain transcription factor heterodimer and its allosteric regulation by DNA binding. FEBS J. 283, 3134–3154 (2016). 27390177 10.1111/febs.13801

[b20] Joshi R. . Functional Specificity of a Hox Protein Mediated by the Recognition of Minor Groove Structure. Cell 131, 530–543 (2007). 17981120 10.1016/j.cell.2007.09.024 PMC2709780

[b21] LaRonde-LeBlanc N. A. & Wolberger C. Structure of HoxA9 and Pbx1 bound to DNA: Hox hexapeptide and DNA recognition anterior to posterior. Genes Dev. 17, 2060–2072 (2003). 12923056 10.1101/gad.1103303 PMC196259

[b22] Mann R. S. & Chan S. K. Extra specificity from extradenticle: the partnership between HOX and PBX/EXD homeodomain proteins. Trends Genet. TIG 12, 258–262 (1996). 8763497 10.1016/0168-9525(96)10026-3

[b23] Passner J. M., Ryoo H. D., Shen L., Mann R. S. & Aggarwal A. K. Structure of a DNA-bound Ultrabithorax-Extradenticle homeodomain complex. Nature 397, 714–719 (1999). 10067897 10.1038/17833

[b24] Foos N. . A Flexible Extension of the Drosophila Ultrabithorax Homeodomain Defines a Novel Hox/PBC Interaction Mode. Structure 23, 270–279 (2015). 25651060 10.1016/j.str.2014.12.011

[b25] Lelli K. M., Noro B. & Mann R. S. Variable motif utilization in homeotic selector (Hox)–cofactor complex formation controls specificity. Proc. Natl. Acad. Sci. 108, 21122–21127 (2011). 22160705 10.1073/pnas.1114118109 PMC3248519

[b26] Sprules T., Green N., Featherstone M. & Gehring K. Lock and key binding of the HOX YPWM peptide to the PBX homeodomain. J. Biol. Chem. 278, 1053–1058 (2003). 12409300 10.1074/jbc.M207504200

[b27] Li T., Stark M. R., Johnson A. D. & Wolberger C. Crystal Structure of the MATa1/MATα2 Homeodomain Heterodimer Bound to DNA. Science 270, 262–269 (1995). 7569974 10.1126/science.270.5234.262

[b28] Mathiasen L., Bruckmann C., Pasqualato S. & Blasi F. Purification and characterization of a DNA-binding recombinant PREP1:PBX1 complex. PloS One 10 (2015). 10.1371/journal.pone.0125789 PMC4391845 25856340

[b29] Yang J. . The I-TASSER Suite: protein structure and function prediction. Nat. Methods 12, 7–8 (2015). 25549265 10.1038/nmeth.3213 PMC4428668

[b30] Penkov D. . Analysis of the DNA-binding profile and function of TALE homeoproteins reveals their specialization and specific interactions with Hox genes/proteins. Cell Rep. 3, 1321–1333 (2013). 23602564 10.1016/j.celrep.2013.03.029

[b31] Dardaei L. . Tumorigenesis by Meis1 overexpression is accompanied by a change of DNA target-sequence specificity which allows binding to the AP-1 element. Oncotarget 6, 25175–25187 (2015). 26259236 10.18632/oncotarget.4488 PMC4694823

[b32] Laurent A. . ChIP-Seq and RNA-Seq Analyses Identify Components of the Wnt and Fgf Signaling Pathways as Prep1 Target Genes in Mouse Embryonic Stem Cells. PloS One 10 (2015). 10.1371/journal.pone.0122518 PMC4395233 25875616

[b33] Abe N. . Deconvolving the recognition of DNA shape from sequence. Cell 161, 307–318 (2015). 25843630 10.1016/j.cell.2015.02.008 PMC4422406

[b34] Panne D., Maniatis T. & Harrison S. C. Crystal structure of ATF-2/c-Jun and IRF-3 bound to the interferon-beta enhancer. EMBO J. 23, 4384–4393 (2004). 15510218 10.1038/sj.emboj.7600453 PMC526468

[b35] Berger M. F. . Variation in homeodomain DNA binding revealed by high-resolution analysis of sequence preferences. Cell 133, 1266–1276 (2008). 18585359 10.1016/j.cell.2008.05.024 PMC2531161

[b36] Thiaville M. M. . Identification of PBX1 target genes in cancer cells by global mapping of PBX1 binding sites. PloS One 7, e36054 (2012). 22567123 10.1371/journal.pone.0036054 PMC3342315

[b37] Ferretti E. . Segmental expression of Hoxb2 in r4 requires two separate sites that integrate cooperative interactions between Prep1, Pbx and Hox proteins. Dev. Camb. Engl. 127, 155–166 (2000). 10.1242/dev.127.1.155 10654609

[b38] Farber P. J. & Mittermaier A. Concerted dynamics link allosteric sites in the PBX homeodomain. J. Mol. Biol. 405, 819–830 (2011). 21087615 10.1016/j.jmb.2010.11.016

[b39] Jabet C., Gitti R., Summers M. F. & Wolberger C. NMR studies of the pbx1 TALE homeodomain protein free in solution and bound to DNA: proposal for a mechanism of HoxB1-Pbx1-DNA complex assembly. J. Mol. Biol. 291, 521–530 (1999). 10448033 10.1006/jmbi.1999.2983

[b40] Sprules T., Green N., Featherstone M. & Gehring K. Conformational changes in the PBX homeodomain and C-terminal extension upon binding DNA and HOX-derived YPWM peptides. Biochemistry (Mosc.) 39, 9943–9950 (2000). 10.1021/bi0001067 10933814

[b41] Wishart D. S. . CS23D: a web server for rapid protein structure generation using NMR chemical shifts and sequence data. Nucleic Acids Res. 36, W496–502 (2008). 18515350 10.1093/nar/gkn305 PMC2447725

[b42] Banerjee-Basu S., Sink D. W. & Baxevanis A. D. The Homeodomain Resource: sequences, structures, DNA binding sites and genomic information. Nucleic Acids Res. 29, 291–293 (2001). 11125116 10.1093/nar/29.1.291 PMC29851

[b43] Moreland R. T., Ryan J. F., Pan C. & Baxevanis A. D. The Homeodomain Resource: a comprehensive collection of sequence, structure, interaction, genomic and functional information on the homeodomain protein family. Database J. Biol. Databases Curation 2009, bap004 (2009). 10.1093/database/bap004 PMC2790301 20157477

[b44] Escalante C. R. . Crystal structure of PU.1/IRF-4/DNA ternary complex. Mol. Cell 10, 1097–1105 (2002). 12453417 10.1016/s1097-2765(02)00703-7

[b45] Klemm J. D. & Pabo C. O. Oct-1 POU domain-DNA interactions: cooperative binding of isolated subdomains and effects of covalent linkage. Genes Dev. 10, 27–36 (1996). 8557192 10.1101/gad.10.1.27

[b46] Xi H. & Blanck G. The IRF-2 DNA binding domain facilitates the activation of the class II transactivator (CIITA) type IV promoter by IRF-1. Mol. Immunol. 39, 677–684 (2003). 12493643 10.1016/s0161-5890(02)00214-6

[b47] Gasteiger E. . Protein identification and analysis tools on the ExPASy Server. John M Walk. Ed Proteomica Protoc. Handb. Humana Press (2005).

[b48] Shevchenko A., Wilm M., Vorm O. & Mann M. Mass spectrometric sequencing of proteins silver-stained polyacrylamide gels. Anal. Chem. 68, 850–858 (1996). 8779443 10.1021/ac950914h

[b49] Rappsilber J., Ishihama Y. & Mann M. Stop and Go Extraction Tips for Matrix-Assisted Laser Desorption/Ionization, Nanoelectrospray, and LC/MS Sample Pretreatment in Proteomics. Anal. Chem. 75, 663–670 (2003). 12585499 10.1021/ac026117i

[b50] Vranken W. F. . The CCPN data model for NMR spectroscopy: development of a software pipeline. Proteins 59, 687–696 (2005). 15815974 10.1002/prot.20449

[b51] Schumann F. H. . Combined chemical shift changes and amino acid specific chemical shift mapping of protein-protein interactions. J. Biomol. NMR 39, 275–289 (2007). 17955183 10.1007/s10858-007-9197-z

[b52] Hafsa N. E., Arndt D. & Wishart D. S. CSI 3.0: a web server for identifying secondary and super-secondary structure in proteins using NMR chemical shifts. Nucleic Acids Res. 43, W370–377 (2015). 25979265 10.1093/nar/gkv494 PMC4489240

