# Supplementary material for: New Insights into Cooperative Binding of Homeodomain Transcription Factors PREP1 and PBX1 to DNA
Source: Sci Rep. 2017 Jan 17;7:40665. doi: 10.1038/srep40665 (PMC5240567; doi:10.1038/srep40665)
Supplement: Supplementary Information and Figures [file srep40665-s1.pdf]

New Insights into Cooperative Binding of Homeodomain Transcription Factors PREP1 and PBX1 to DNA

Chiara Zucchelli, Elena Ferrari, Francesco Blasi, Giovanna Musco, Chiara Bruckmann

SUPPLEMENTARY INFORMATION

Oligo	Sequence 5'-3'	Restriction site
**PBX1<227**	CGCGGATCCATGCGTTCCCGATTTCTGG	BamHI
**Prep1>344**	CCGCTCGAGTTACCTCTGAACTGGCCGGTTC	XhoI
**Prep1<257**	CCGGGATCCATGGGTTCATCTAAGAACAAGAGGGGC	BamHI
**Prep1>325**	CCGCTCGAGTTAACTTGAATCCAACATTGGCTGAAGA	XhoI
**HOXB1<171**	CCGGGATCCATGGAACCTAACACCCCCACGGCC	BamHI
**HOXB1>266**	CCGCTCGAGTTAACCTCCCTCTCGCTCGCG	XhoI
**PREP1 240>**	CGCGGATCCATGCAGCTTCAGTTACAGTTAAACCAAGA	BamHI
**PREP1 > 257**	CGCGGATCCATGGGTTCATCTAAGAACAAGAGGGG	BamHI
**Prep1 < 325**	GCGCTCGAGTTAAACTTGAATCCAACATTGGCT	XhoI

Table S1: DNA primers used for cloning PREP1$_{HD}$, PBX1$_{HD}$, PREP1$_{hd}$ and HOXB1$_{HD}$ constructs.

	mW (Da)	Abs 0.1% (=1 g/l)
**PREP1 $_{HD}$**	12168	1.026
**PREP1 $_{hd}$**	7983	1.565
**PREP1 $_{hd-N}$**	9986	1.251
**PREP1 $_{hd-C}$**	10165	1.229
**PBX1$_{HD}$**	10465	1.237
**HOXB1$_{HD}$**	10840	1.156

Table S2: Parameters calculated by means of the ExPASy ProtParam tool (http://web.expasy.org/protparam/) and used for UV concentration determination

Figure S1

Panel A: SDS PAGE of PREP1 HDs, after one purification step (GST affinity chromatography). Lane 1: PREP1$_{HD}$; lane 2: PREP1$_{hd}$; lane 3: PREP1$_{hd-N}$; PREP1$_{hd-C}$.

Panel B: Last step of purification of PREP1$_{HD}$. On the left, gel filtration profile of PREP1$_{HD}$ (in blue) compared to molecular weight standards (in green). On the right, the SDS PAGE gel of the eluted protein.

Panel C: Last step of purification of PREP1$_{hd}$. On the left, gel filtration profile of PREP1$_{hd}$ (in blue) compared to molecular weight standards (in green). On the right, the SDS PAGE gel of the eluted protein.

Figure S2

EMSA titration of PREP1$_{hd-N}$ (**Panel A**) and PREP1$_{hd-C}$ (**Panel B**) with PMH oligo. Protein:DNA ratios were 0.5 (DNA excess), 1 (equimolar ratio), or 2 (protein excess). Gels were stained both with ethidium bromide and coomassie blue to visualize DNA or proteins respectively.

Panel C: Peptides of PREP1$_{HD}$ and PBX1$_{HD}$ identified by mass spectrometry (in red) in the band purified band from the EMSA titration of **Figure 3A**.

Panel D: Peptides of HOXB1$_{HD}$ and PBX1$_{HD}$ identified by mass spectrometry (in red) in the purified band from the EMSA titration shown in **Figure 3E**.

Figure S3

Panel A: EMSA with single HoxB1. DNA probes were incubated with different amounts of HoxB1. Protein:DNA ratios were 0.5 (DNA excess), 1 (equimolar ratio), or 2 (protein excess). Gels were stained both with ethidium bromide and coomassie blue to visualize DNA or proteins respectively. HOXB1$_{HD}$ binds all three DNA probes. Sharp bands in the control titration indicate that the HOXB1$_{HD}$ monomer binds also the control DNA.

Panel B: K_D values for individual HoxB1 with different DNA sequences, measured by fluorescence polarization. The values are the average of three separate experiments (n=3), each run in triplicate

Panel C: Fluorescence polarization K_D measurement of PREP1 HD in the presence of a preformed HOXB1 HD:DNA complex, and of HoxB1 HD in the presence of a preformed PBX1$_{HD}$: DNA complex. DNA sequences used: PMH (binding site of PBX1:PREP1), PH (binding site of PBX1:HOXB1), and an aspecific DNA sequence as control. The values are the average of three separate experiments (n=3), each run in triplicate.

Panel D: Titration of $PREP1_{HD}$ with $HOXB1_{HD}$:PMH complex. PMH and $HOXB1_{HD}$ at a fixed concentration (8μM and 4 μM, respectively) were titrated with increasing $PREP1_{HD}$ (2, 4 and 8 μM). The formation of the heterodimer $PREP1_{HD}$-HOXB1 complex is not favored, because no retarded band is visualized (lanes 3-5), whereas the free DNA pool decreases, as visible at the bottom of the gel.

Panel E: Titration of $HOXB1_{HD}$:PMH with $PREP1_{hd}$. PMH oligo and $HOXB1_{HD}$ at a fixed concentration (8μM and 4 μM respectively) were titrated with increasing concentrations of $PREP1_{hd}$ (2, 4 and 8μM). Upon increase of $PREP1_{hd}$ no retarded band above the individual PMH:$PREP1_{hd}$ and PMH:$HOXB1_{HD}$ complexes (lanes 3-5) are observed.

Panel F: Titration of PMH:$PBX1_{HD}$ with $HOXB1_{HD}$. PMH and $PBX1_{HD}$ at a fixed concentration (8μM and 4 μM, respectively) were titrated with increasing amounts of $HOXB1_{HD}$ (2, 4 and 8μM). The band, marked with a star, in lane 5, was analyzed by mass spectrometry.

Panel G: Mass spectrometry analysis of EMSA titrations band from Panel F of this Figure. The slow-migrating band indicated with red asterisks in **Panel F** was cut and analyzed by mass spectrometry (see Methods). The band contains both PBX1 and HOXB1 HDs. Gi (sequence identification number), number of unique identified peptides and their relative Mascot score are shown for each protein.

Figure S4

Circular dichroism (CD) spectra of $PREP1_{HD}$ (magenta) and $PREP1_{hd}$ (blue) recombinant proteins (MRE, molar residue ellipticity)

Figure S5

Panel A: Secondary structure prediction and chemical shift index (CSI) plot for $PREP1_{HD}$ calculated by CSI 3.0. The shorter $PREP1_{hd}$ sequence, for which an NMR structure has been deposited (1X2N.pdb), is highlighted in blue.

Panel B: Superposition of $PREP1_{HD}$ structural model (generated by CSD23D web server) in grey with the $PREP1_{hd}$ structure (1X2N.pdb) in blue. (RMS 0.20 Å on CA atoms residues 272-330)

Figure S6

^{15}N HSQC spectra acquired during the NMR titration of ^{15}N-labeled $PREP1_{HD}$ with the PMH DNA oligo.

Figure S7

^{15}N HSQC spectra acquired during the NMR titration of ^{15}N-labeled PREP1$_{hd}$ with the PMH DNA oligo.

Figure S8

Plot showing the combined chemical shift displacement (CSD) of the backbone NH groups observed upon PMH addition to ^{15}N-labeled PREP1$_{hd}$ (blue) or PREP1$_{HD}$ (magenta), at 1:0.5 (protein:PMH RATIO). Horizontal lines indicate average plus standard deviation.

Figure S1

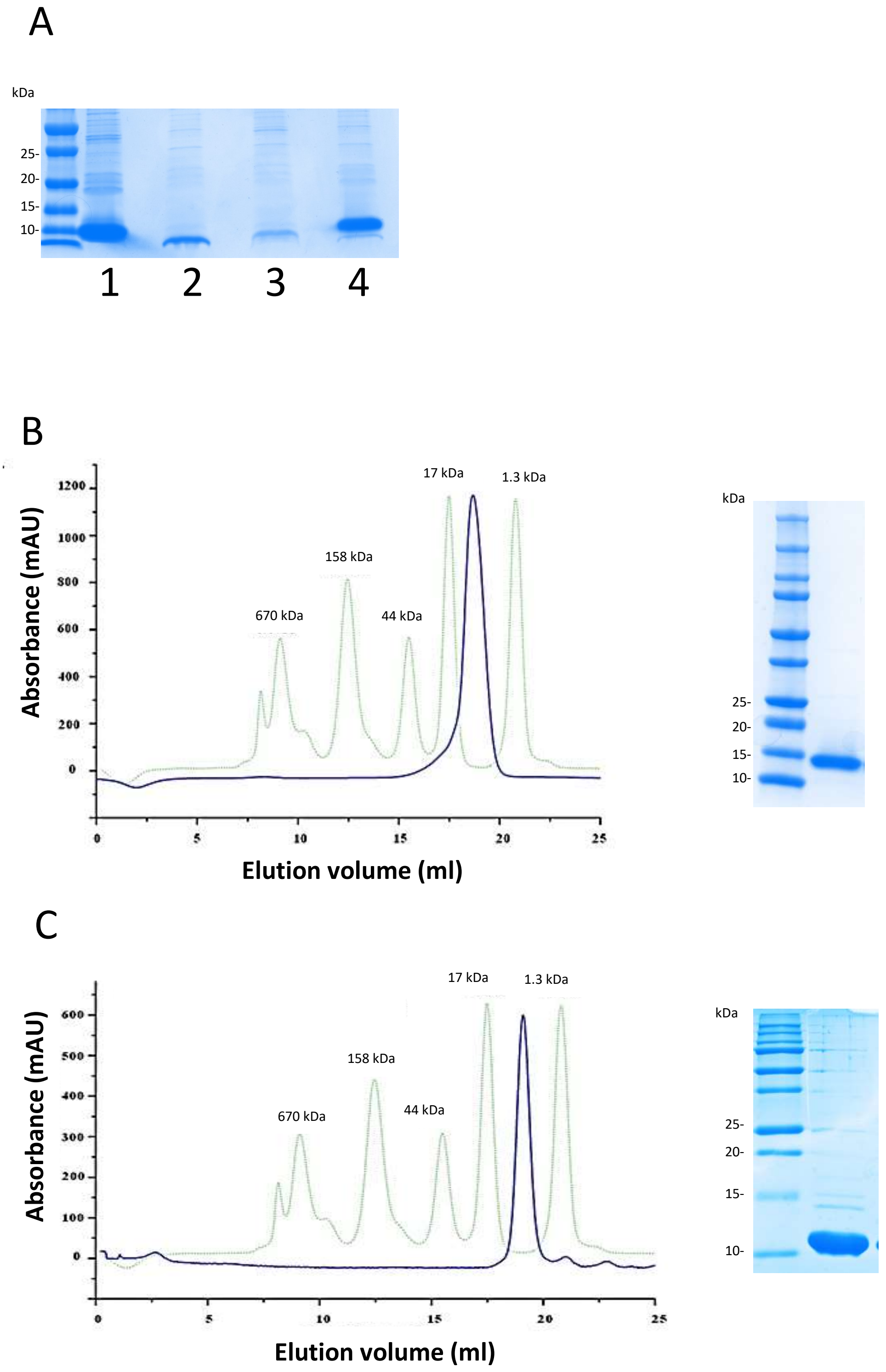
A
kDa
25-
20-
15-
10-
1
2
3
4
B
Absorbance (mAU)
1200
1000
800
600
400
200
0
670 kDa
158 kDa
44 kDa
17 kDa
1.3 kDa
0
5
10
15
20
25
Elution volume (ml)
kDa
25-
20-
15-
10-
C
Absorbance (mAU)
600
500
400
300
200
100
0
670 kDa
158 kDa
44 kDa
17 kDa
1.3 kDa
0
5
10
15
20
25
Elution volume (ml)
kDa
25-
20-
15-
10-

Figure S2

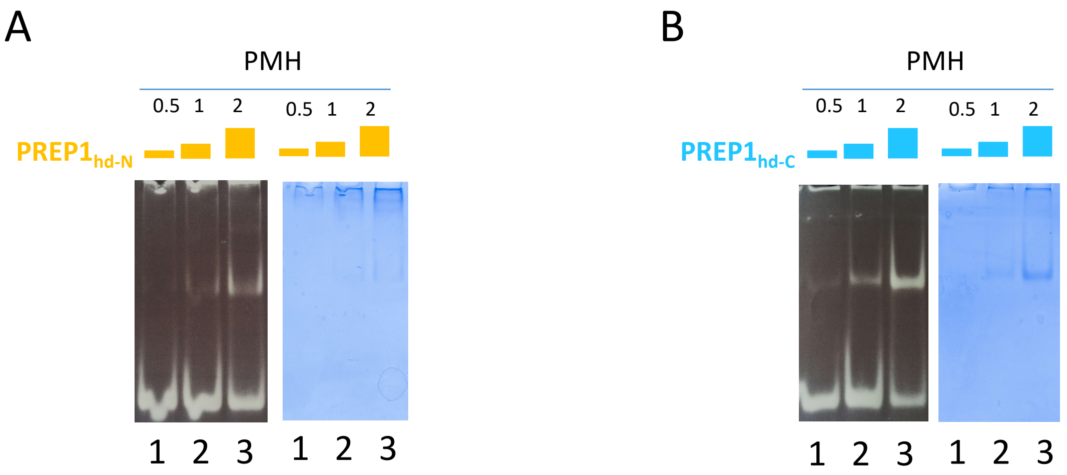

C

gi|2052385 - PREP1
240-QLQLQLNQDLSILHQDDGSSKNKRGVLPKHATNVMRSWLFQHIGHPYPTEDEKKQIAAQTN
LTLLQVNNWFINARRRILQPMLDSSCSETPKTKKKTAQNRPVQRF-344

gi|107390 - PBX1
227-SRFLDARRKRRNFNKQATEILNEYFYSHLSNPYPSEEAKEELAKKCGITVSQVSNWFGNKRIRYK
KNIGKFQEEANIYAAKTAVTATNVS-317

Figure S3

A

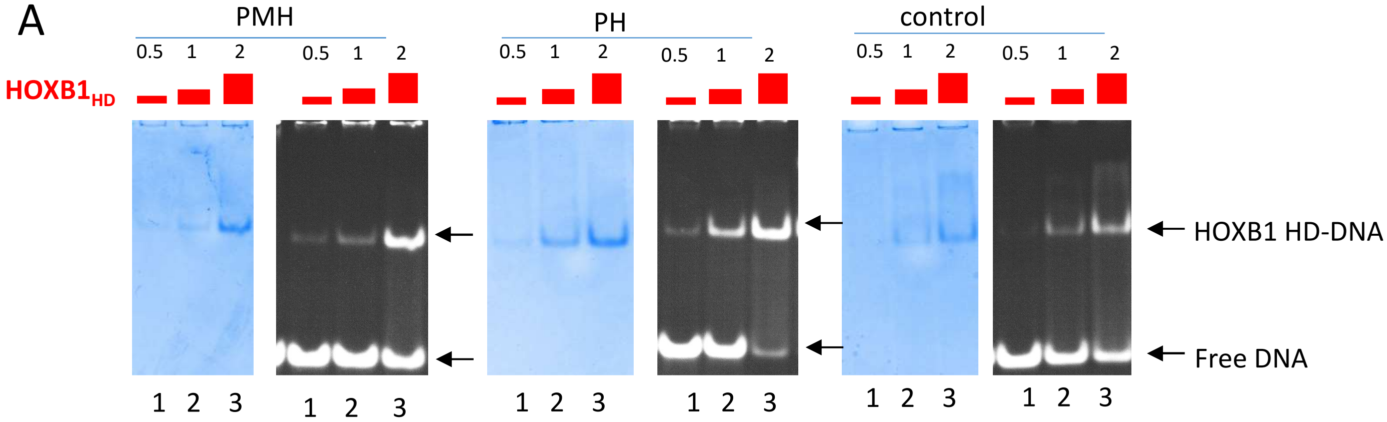

B

oligo	K_D (µM)
PMH	2.7± 0.2
PH	6.2 ± 0.1
Control	24.7 ± 0.2

C

Proteins	K_D (µM)
PREP1$_{HD}$ + HOXB1$_{HD}$:PMH oligo	13.0 ± 2.1
PREP1$_{hd}$+ HOXB1$_{HD}$:PMH oligo	34.7 ± 1.1
HOXB1$_{HD}$ + PBX1$_{HD}$:PMH oligo	1.3 ± 0.3
HOXB1$_{HD}$+ PBX1$_{HD:}$control oligo	34.7 ± 1.7
HOXB1$_{HD}$+ PBX1$_{HD}$:PH oligo	0.39 ±0.1

D

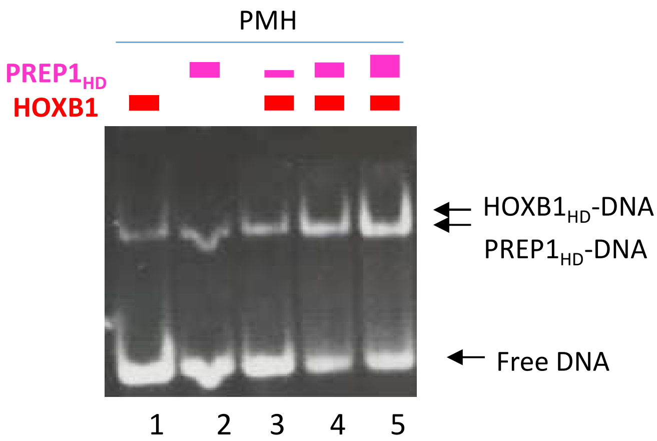

E

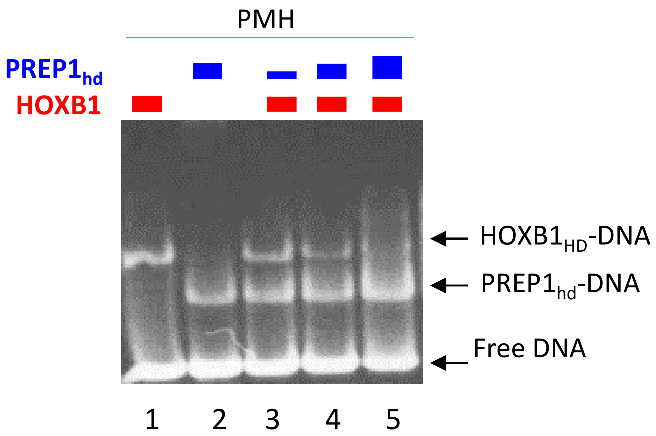

F

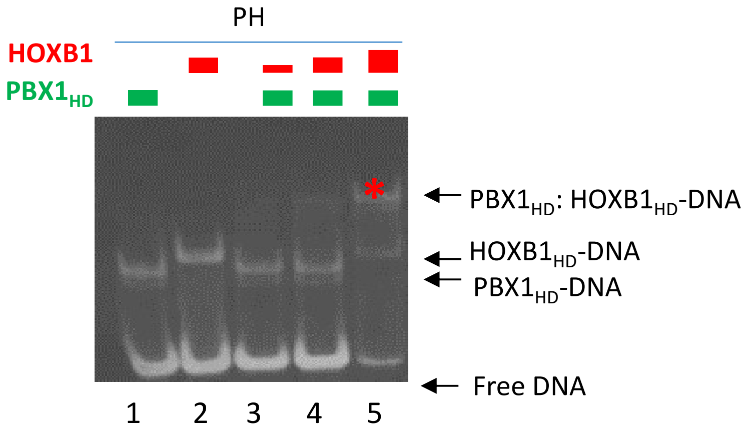

G

Gi	Protein	Identified peptides	Mascot score
gi|32384	HOXB1	6	420
gi|107390	PBX1	5	257

Figure S4

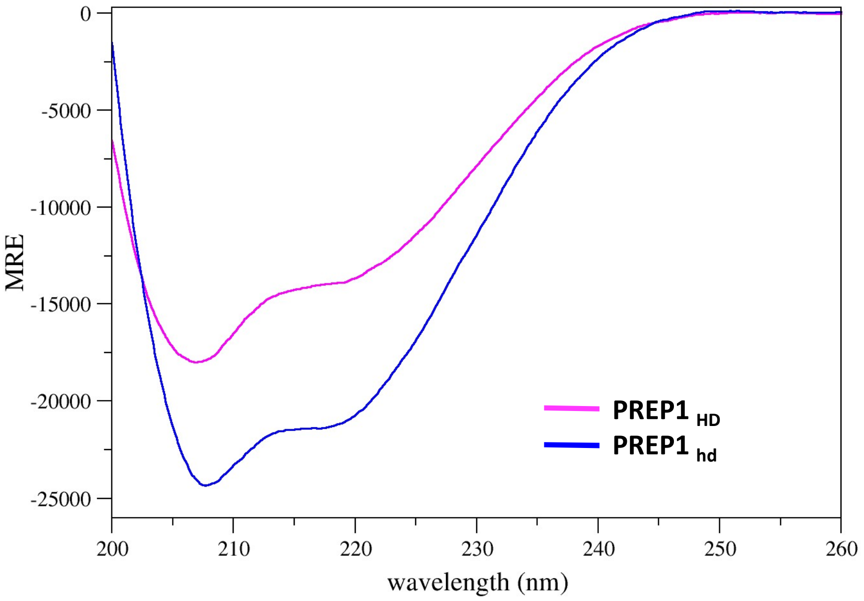

0
-5000
-10000
-15000
-20000
-25000
MRE
200
210
220
230
240
250
260
wavelength (nm)
PREP1 HD
PREP1 hd

Figure S5

A

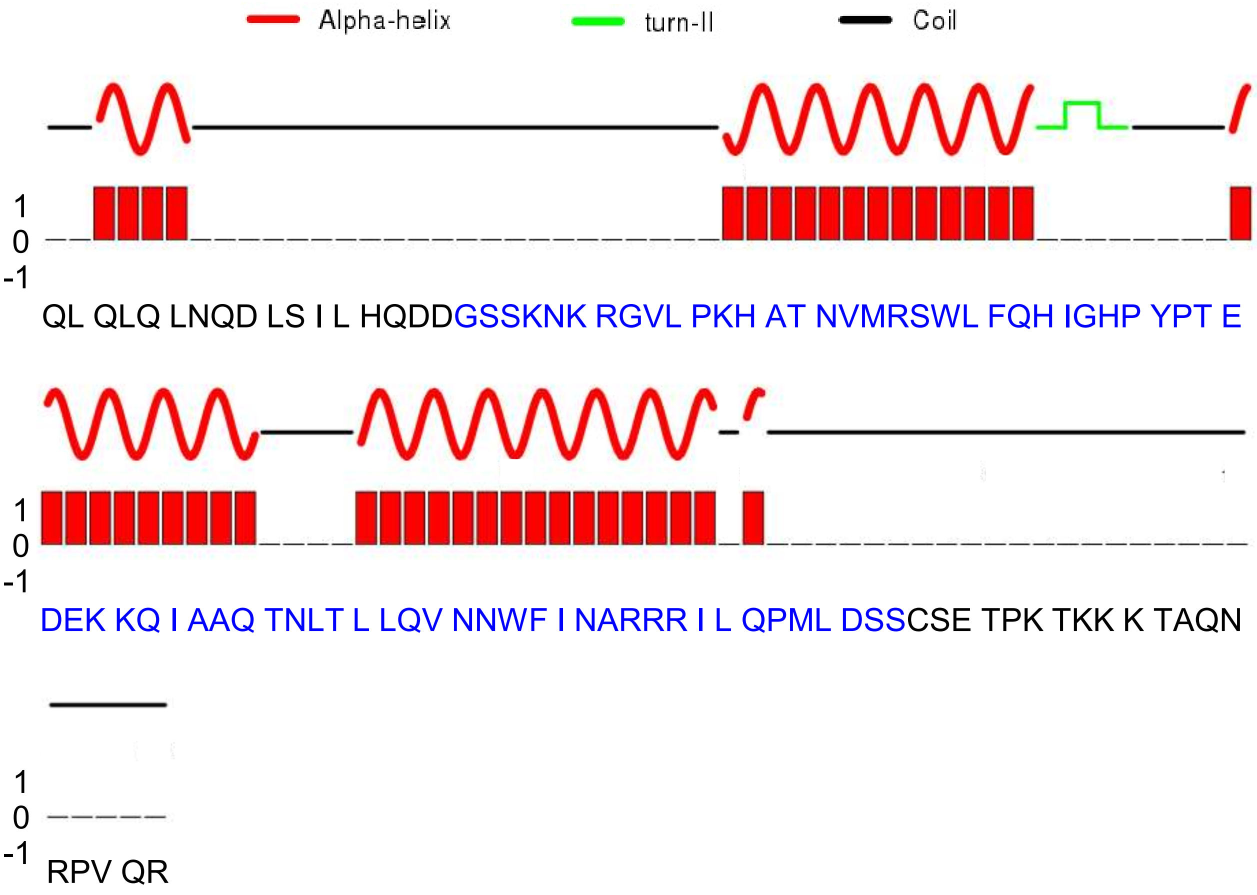

Alpha-helix
turn-II
Coil
1
0
-1
QL QLQ LNQD LS I L HQDDGSSKNK RGVL PKH AT NVMRSWL FQH IGHP YPT E
1
0
-1
DEK KQ I AAQ TNLT L LQV NNWF I NARRR I L QPML DSSCSE TPK TKK K TAQN
1
0
-1
RPV QR

B

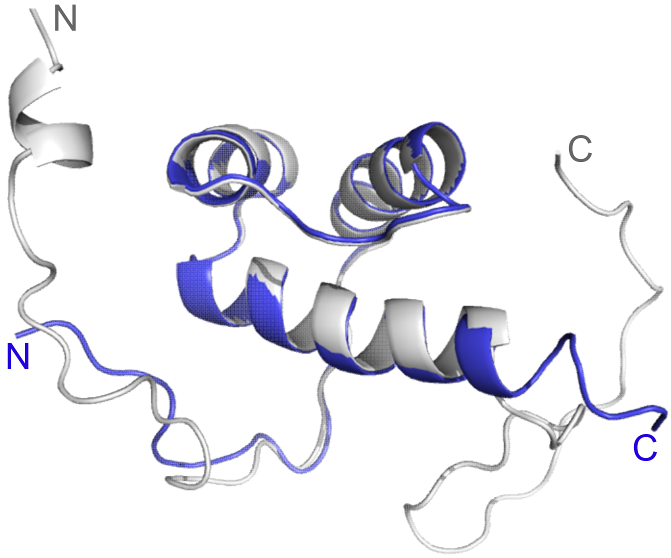

N
C
N
C

Figure S6

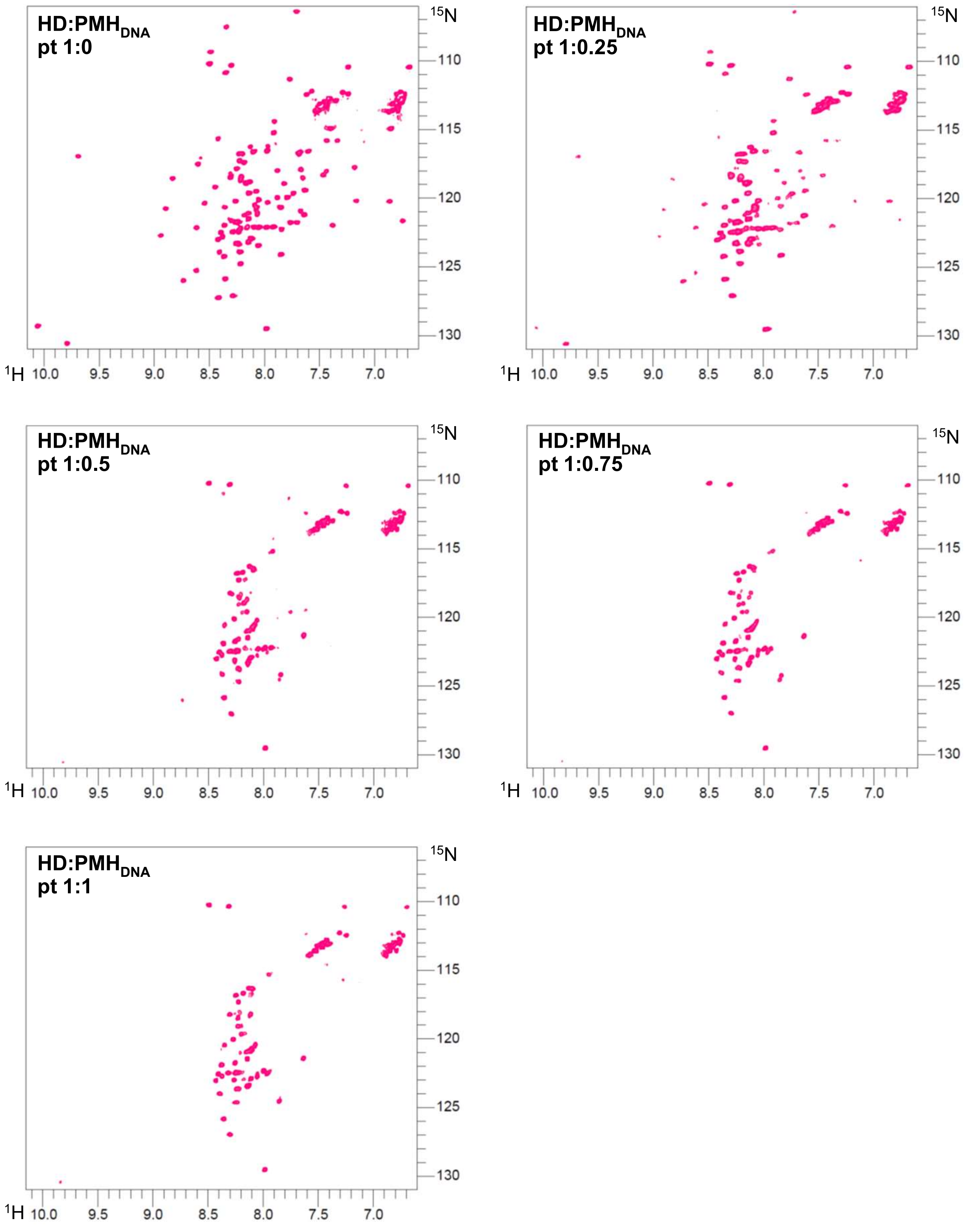
HD:PMH_{DNA}
pt 1:0
HD:PMH_{DNA}
pt 1:0.25
HD:PMH_{DNA}
pt 1:0.5
HD:PMH_{DNA}
pt 1:0.75
HD:PMH_{DNA}
pt 1:1
^{15}N
110
115
120
125
130
^{1}H
10.0
9.5
9.0
8.5
8.0
7.5
7.0

Figure S7

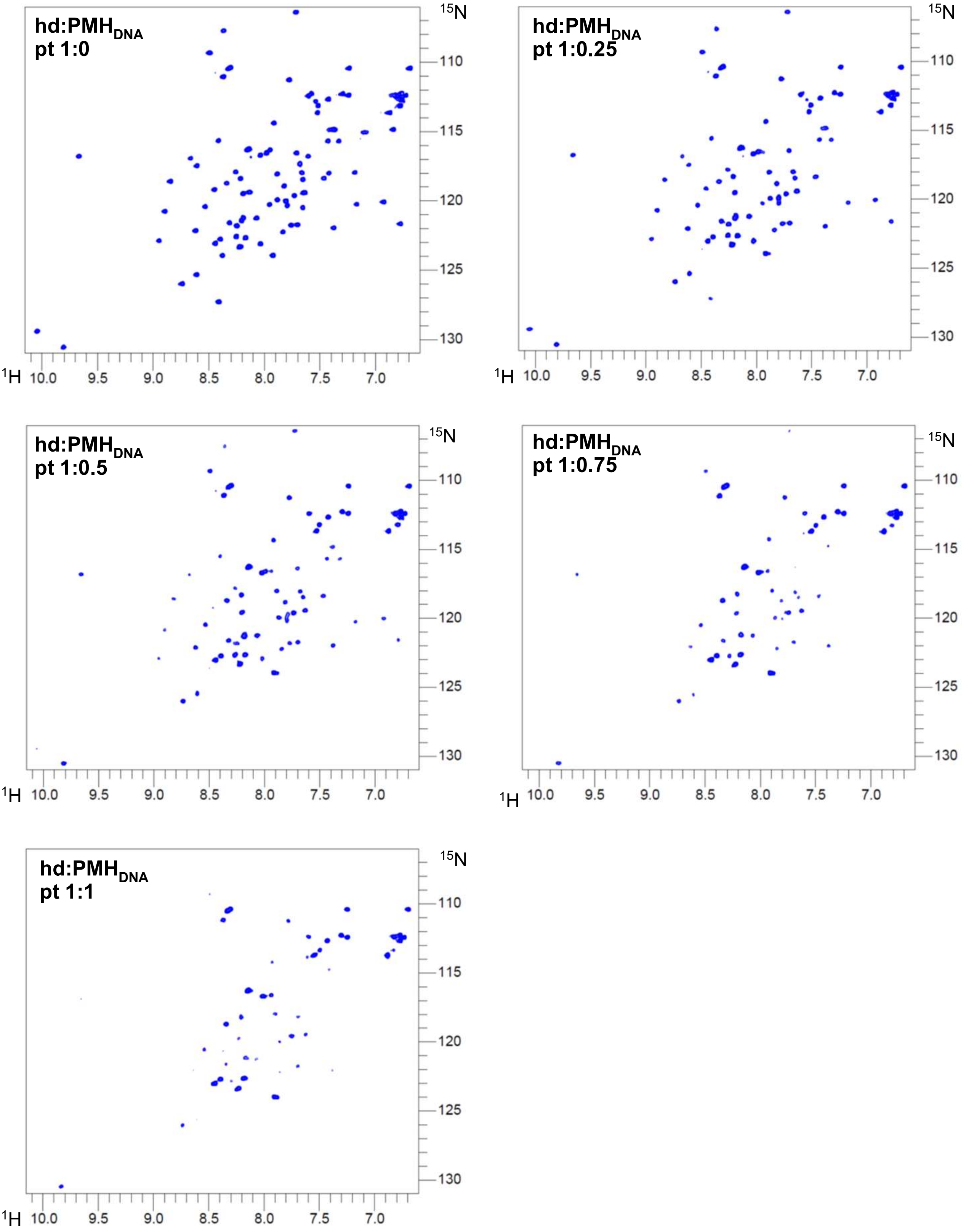

hd:PMH$_{DNA}$
pt 1:0
hd:PMH$_{DNA}$
pt 1:0.25
hd:PMH$_{DNA}$
pt 1:0.5
hd:PMH$_{DNA}$
pt 1:0.75
hd:PMH$_{DNA}$
pt 1:1
^{15}N
110
115
120
125
130
^{1}H
10.0
9.5
9.0
8.5
8.0
7.5
7.0

Figure S8

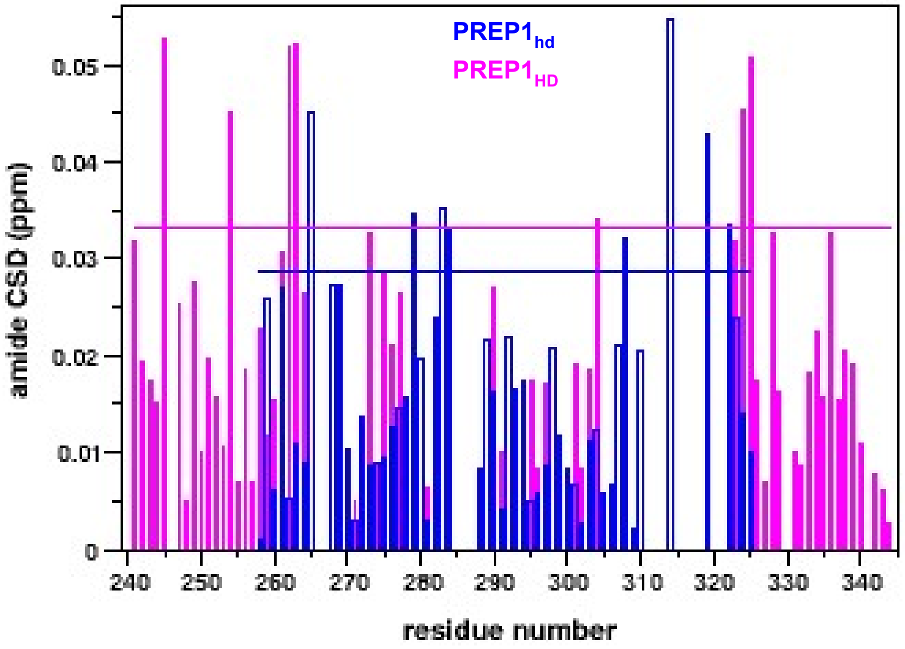
PREP1hd
PREP1HD
amide CSD (ppm)
0.05
0.04
0.03
0.02
0.01
0
240
250
260
270
280
290
300
310
320
330
340
residue number
